# Dual roles of CD11b^+^CD33^+^HLA-DR^-/low^CD14^-^ myeloid-derived suppressor cells with a granulocytic morphology following allogeneic hematopoietic stem cell transplantation: from inflammation promoters to immune suppressors within 90 days

**DOI:** 10.3389/fimmu.2024.1403272

**Published:** 2024-07-08

**Authors:** Ming Ni, Jing Cui, Xin Yang, Yuntian Ding, Peng Zhao, Tianzhen Hu, Yun Zhan, Qian Kang, Xiuying Hu, Jiangyuan Zhao, Yao Xu, Lu Chen, Min Liu, Mei Zhao, Fengqi Zhang, Shisi Huang, Ya Li, Xueying Yang, Luxin Zhang, Tianzhuo Zhang, Bo Deng, Bing Yang, Deqin Lu, Jishi Wang

**Affiliations:** ^1^ Department of Hematology, Affiliated Hospital of Guizhou Medical University, Guiyang, China; ^2^ Department of Dermatology, Affiliated Hospital of Guizhou Medical University, Guiyang, China; ^3^ Department of Hematology, The Second Affiliated Hospital of Guizhou Medical University, Kaili, China; ^4^ Department of Internal Medicine V, University Clinic Heidelberg, Heidelberg, Germany; ^5^ Department of Pathophysiology, Guizhou Medical University, Guiyang, China

**Keywords:** G-MDSCs, allo-HSCT, aGVHD, HO-1, ER-stress, immunomodulation, T cells

## Abstract

**Introduction:**

Granulocytic myeloid-derived suppressor cells (G-MDSCs) show fast recovery following allogeneic hematopoietic stem cell transplantation (allo-HSCT) constituting the major part of peripheral blood in the early phase. Although G-MDSCs mediate immune suppression through multiple mechanisms, they may also promote inflammation under specific conditions.

**Methods:**

G-MDSCs were isolated from 82 patients following allo-HSCT within 90 days after allo-HSCT, and their interactions with autologous CD3^+^ T-cells were examined. T-cell proliferation was assessed by flow cytometry following CFSE staining, while differentiation and interferon-γ secretion were characterized using chemokine receptor profiling and ELISpot assays, respectively. NK cell cytotoxicity was evaluated through co-culture with K562 cells. An aGVHD xenogeneic model in humanized mice was employed to study the in vivo effects of human leukocytes. Furthermore, transcriptional alterations in G-MDSCs were analyzed via RNA sequencing to investigate functional transitions.

**Results:**

G-MDSCs promoted inflammation in the early-stage, by facilitating cytokine secretion and proliferation of T cells, as well as their differentiation into pro-inflammatory T helper subsets. At day 28, patients with a higher number of G-MDSCs exhibited an increased risk of developing grades II-IV aGvHD. Besides, adoptive transfer of G-MDSCs from patients at day 28 into humanized mice exacerbated aGvHD. However, at day 90, G-MDSCs led to immunosuppression, characterized by upregulated expression of indoleamine 2,3-dioxygenase gene and interleukin-10 secretion, coupled with the inhibition of T cell proliferation. Furthermore, transcriptional analysis of G-MDSCs at day 28 and day 90 revealed that 1445 genes were differentially expressed. These genes were associated with various pathways, revealing the molecular signatures of early post-transplant differentiation in G-MDSCs. In addition, genes linked to the endoplasmic reticulum stress were upregulated in patients without aGvHD. The acquisition of immunosuppressive function by G-MDSCs may depend on the activation of *CXCL2* and *DERL1* genes.

**Conclusion:**

Our findings revealed the alteration in the immune characteristics of G-MDSCs within the first 90 days post-allo-HSCT. Moreover, the quantity of G-MDSCs at day 28 may serve as a predictive indicator for the development of aGvHD.

## Introduction

Myeloid-derived suppressor cells (MDSCs) are a mixture of immature myeloid cells (1). Generally, they are defined as cells with a phenotype of CD11b^+^CD33^+^HLA-DR^-/low^ among peripheral blood mononuclear cells (PBMCs) isolated after density gradient, and could be further classified into monocytic MDSCs (M-MDSC) and granulocytic MDSCs (G-MDSC) based on the expression of CD14 and CD15, respectively (2–6). The role of MDSCs in immune responses is still debated. Although it is well known that the most striking functional feature that defines MDSCs is their ability to suppress T cell-mediated immune responses (7), several studies have reported that situations in which MDSCs can exacerbate inflammation (1, 8, 9). In a murine sepsis model, Gazar et al. showed that MDSCs promoted the inflammation in the early stage, while secreted anti-inflammatory cytokines in the later phase, indicating MDSCs may become immunosuppressive under prolonged stimuli (9). Also, an increased number of MDSCs has been associated with unfavorable outcomes in patients with sepsis (10).

In allogeneic hematopoietic stem cell transplantation (allo-HSCT), G-MDSCs in graft have been reported to offer protection against acute graft versus host disease (aGvHD). Huang et al. demonstrated that HLA-DR^-/low^CD33^+^CD16^-^ MDSCs in the graft prevented aGvHD in humanized mice, and have a significant role in reducing the incidence of GvHD in patients following allo-HSCT (11). On the other hand, Cuvelieret et al. showed that patients with a higher number of G-MDSCs at preconditioning suffer from a greater risk of aGvHD (12). After engraftment, immunomonitoring suggested an early recovery of G-MDSCs (12, 13). Nevertheless, further relevant function assays are often lacking due to their short lifespan and inability to cryopreserve or expand *in vitro* (14). Hence, the data is primarily derived from preclinical research. The findings of these investigations indicate that G-MDSCs in graft could prevent aGvHD. However, the function of G-MDSCs during the immune reconstitution phase remains controversial (7). Furthermore, these studies cannot fully simulate the conditions within patients, especially in the case of patients undergoing long-term treatment with immunosuppressive medications.

Heme oxygenase-1 (HO-1) has a critical role in allo-HSCT by mitigating oxidative stress and reducing the incidence of aGVHD. Studies have shown that HO-1 facilitates the maintenance of redox homeostasis and diminishes cellular oxidative damage, significantly decreasing the severity of aGVHD (15–17). Inducing HO-1 expression prior to transplantation has been shown to enhance survival rates and alleviates the symptoms of aGVHD, demonstrating its potential therapeutic benefits (16). Additionally, the expression level of donor-derived myeloid HO-1 is crucial in preventing lethal experimental GVHD (17). Therefore, monitoring HO-1 expression in G-MDSCs may serve as a predictive marker for changes in oxidative stress status and the onset of aGVHD.

In this study, we analyzed the kinetics of G-MDSCs recovery after transplantation and their characteristics. Moreover, in contrast to previous studies that consider G-MDSCs as low-density neutrophils (18), recent publications have revealed that the immunosuppressive role of neutrophils/G-MDSCs does not always correlate with their density (19, 20). Therefore, we obtained all leukocytes rather than selectively isolating those with low density. Based on the clinical samples, function assays, humanized mice, RNA sequencing and genetic overlap analysis, we demonstrated the features of CD11b^+^CD33^+^HLA-DR^-/low^CD14^-^ MDSCs with a granulocytic morphology, shifting from the immunostimulation to immunosuppression within 90 days post-transplantation.

## Materials and methods

### Healthy donor samples

Healthy donors were recruited from the matched donors of patients who underwent haplo-identical or HLA-matched allo-HSCT, at affiliated hospital of Guizhou Medical University from December 2022 to September 2023. Peripheral blood samples from donors treated with recombinant human G-CSF were collected. Informed consent was obtained before collection. The study was approved by the Ethics Committee of Guizhou Medical University.

### Patients and treatment

A total of 182 samples from 82 patients who underwent allo-HSCT in affiliated hospital of Guizhou Medical University from September 2020 to November 2023 were analyzed. The inclusion criteria were: (i) Between 14 and 60 years old; (ii) Acute myeloid leukemia (AML) patients in the first complete remission (CR1) identified as intermediate to high risk, or all patients in CR2; (iii) Acute lymphoblastic leukemia (ALL) patients in CR1; (iv) Myelodysplastic syndromes (MDS) patients categorized as having an intermediate to high international prognostic scoring system (IPSS) score; (v) Chronic myelogenous leukemia (CML) patients with the T315I mutation or who have experienced the accelerated or blast phase; (vi) Non-Hodgkin lymphoma patients with poor initial treatment responses, refractory conditions, or relapses; (vii) Patients diagnosed with severe aplastic anemia (SAA) or very severe SAA (vSAA); (viii) Hemophagocytic syndrome patients.

The optimal donor is a sibling with a complete HLA match. In the absence of a suitable sibling, options include haploidentical relatives or unrelated volunteer donors. For haploidentical donors, the preferred order is male siblings, followed by female siblings, children, fathers, mothers, and other collateral relatives. High-resolution typing of HLA-A, B, C, DRB1, and DQ is required for unrelated volunteers. Preferred matches are either 10/10 or 9/10, with a secondary preference for 8/10 matches. These matches must have a minimum compatibility of 5/6 genetic locations for HLA-A, B, and DRB1.

The collection of patient specimens was structured into 2 phases. Patients with failed implantations were excluded from the study. In Phase 1, peripheral blood specimens from 29 patients were analyzed for MDSC levels at 6 distinct time points post-transplantation. Phase 2 involved 53 additional patients from various time points to explore two aspects: verifying the correlation between MDSC levels at day 28 and the onset of aGvHD, as well as conducting functional assays, animal studies, and sequencing.

All patients and/or their relatives signed the informed consent under the frame of the declaration of Helsinki. The study was approved by the Ethics Committee of Guizhou Medical University. Demographics and outcomes of patients are presented in **Supplementary Table 1**. Further details are available in **Supplementary information**.

### T-cell proliferation assay

Cells were obtained from autologous or related donors to prevent allogeneic reactions CD3^+^ T cells were purified by negative selection via flow cytometry, followed by labeling with 5 μM 5,6-carboxy-fluorescein diacetate succinimidyl ester (CFSE) (Biolegend) and co-culturing with G-MDSCs at a ratio of 1:1. Cells were cultured in 96-well U bottom plate with pre-coated anti-CD3/CD28 antibodies (Biolegend) in RPMI 1640 (Bio-Channel), supplemented with 10% FBS (SORFA) and 2mM L-glutamine for 4 days. After incubation, cells were stained with CD8 phycoerythrin (PE) (Clone: 4AHT8a) (4A Biotech) and CD4 allophycocyanin (APC) (Clone: 13B8.2) (BECKMAN COULTER). 7AAD was used to exclude dead cells. All acquisitions were performed on a FACSLyric FACS device (BD biosciences).

### T-cell differentiation assay

CD3^+^ T cells were obtained and co-cultured as described above without CFSE staining. After 4 days, T cells were stained with CD4 brilliant violet 510 (BV510) (Clone: RPA-T4)(Biolegend), CD8 allophycocyanin-cyanine7 (APC-Cy7) (Clone: SK1) (Biolegend), CD45RA allophycocyanin-eFluor 780 (Alexa Fluor 700) (Clone: HI100) (Biolegend), CD183 fluorescein isothiocyanate (FITC) (Clone: G025H7) (Biolegend), CD194 APC (Clone: L291H4) (Biolegend), CD196 phycoerythrin-cyanin 7 (PE-Cy7) (Clone: G034E3) (Biolegend), CD185 brilliant violet 421 (Clone: J252D4) (BV421) (Biolegend) and CCR10 PE (Clone: May-88) (Biolegend). The percentages of CD4^+^ T cell subsets were detected via flow cytometry according to the expression of chemokine receptors (21).

### T-cell interferon-γ release assay

As described above, 1x10^5^ freshly sorted G-MDSCs were mixed with CD3^+^ T cells at a ratio of 1:1. CMV peptide pool (MABTECH) was added directly into the experimental wells. The enzyme-linked immunosorbent spot (ELISpot) assay was conducted following the manufacturer’s instructions. Image analysis of ELISpot plates was performed with an AID ELISpot reader (Autoimmun Diagnostika GmbH).

### Fluorescence activated cell sorting of granulocytic myeloid-derived suppressor cells

Fresh blood samples collected for leukocyte isolation are processed within 2 hours to improve leukocyte separation efficiency. Leukocytes were isolated using Polymorphprep (Serumwerk Bernburg AG) before sorting. After isolation, G-MDSCs were sorted by FACSMelody (BD biosciences) using CD11b PE (Clone: Bear1) (BECKMAN COULTER), CD33 FITC (Clone: D3HL60.251) (BECKMAN COULTER), HLA-DR Peridinin chlorophyll protein complex-Cyanin 5.5 (PerCP-Cy5.5) (Clone: G46-6) (BD biosciences), and CD14 Per-CP-Cy5.5 (Clone: M5E2) (BD biosciences).

### Flow cytometry

Immunomonitoring was performed on freshly collected samples. To optimize cell viability, the period from sample acquisition to staining was less than 2 hours. Antibodies have been summarized in **Supplementary Table 2**. The mean fluorescence intensity (MFI) error between different days was corrected by the MFI of Precision Counting Beads (Biolegend). Briefly, cells were blocked using 5% BSA (absin) containing PBS (absin) for 10 min and stained with CD33 Alexa Fluor 700 (Clone: WM53) (BD), CD14 APC (Clone: 61D3) (ThermoFisher), HLA-DR APC-eFluor780 (Clone: LN3) (ThermoFisher), and CD11b PerCP-Cy5.5 (LM2) (Biolegend) for 20 min in the dark, followed by washing once with FACS buffer (1% BSA and 2 mM ethylenediaminetetraacetic acid in PBS). Afterwards, erythrocytes were lysed using FACS lysing Solution (BD Biosciences). For intracellular staining, cells were fixed and permeabilized using BD FACS Permeabilizing Solution 2 (BD biosciences), then finally stained with HO-1 FITC (Clone: HO-1-2) (abcam) and IL-10 PE-Cy7 (JES3-9D7) (Biolegend) antibodies for 30 min. Precision Counting Beads were added into the sample before acquisitions. All acquisitions were performed on a FACSLyric FACS device. Data were analyzed using Flowjo software (BD biosciences).

### NK cytotoxicity assay

The assessment of NK cytotoxicity was performed as described previously (22). Briefly, 5 x 10^5^ freshly obtained PBMCs from patients were co-cultured with 5 × 10^4^ K562 cells in the presence of CD107a antibody for 6 h. After the first hour, 1 μl of 100X monensin (MultiSciences) and brefeldin A (MultiSciences) was added to the mix.

### Xenogeneic model of graft versus host disease

Six to 7 weeks old male NOD/ShiLtJGpt-*Prkdcem26Cd52Il2rgem26Cd22*/Gpt (NCG) mice were purchased from GemPharmatech (GemPharmatech). All mice were housed 5 per cage in specific pathogen-free facility microisolator cages, and utilized at 8 to 12 weeks old in protocols approved by the local Ethics Committee.

NCG mice were irradiated with 150cGy X-ray at day -1 and PBMCs from patient matched donor were thawed. Following thawing and washing, the resting procedure of cells has been performed according to the literature (23). PBMCs were resuspended in culture medium at 2 x 10^6^ cells/ml, not exceeding 10 ml in 50 ml Falcon tubes. Afterwards, tubes were incubated at 37°C with 5% CO_2_ for 18 hours, tilted at a 5° angle. To allow air exchange, lids on the tubes were slightly loosened. After incubation, cells underwent a second wash in the same medium. Dead cell clumps were filtered out using a 40µm mesh to prevent embolism in mice, and cell counts were performed prior to the experiments. Viability was assessed using Trypan blue.

At day 1, 1 × 10^6^ G-MDSCs from patients and 5 × 10^6^ PBMCs from the related donor of patient were injected into the tail vein. The viability of PBMCs were higher than 70%. Engraftment of human leukocytes was monitored every 7 days and Th subsets were detected once the human CD3^+^ T cells exceeded 20%. The body weight and health status of mice were monitored every 2 days. Mice were sacrificed at day 42 or euthanized if the following criteria are achieved: 1. Weight loss is higher than 15%; 2. Healthy score is higher than 3, or 3. Cumulative healthy score is ≥ 6 (24). Tissues were kept in 4% paraformaldehyde, and paraffin sections were stained with hematoxylin, eosin, and safran. A smart digital camera pannoramic MIDI (3DHISTECH) mounted on a Nikon ECLIPSE E100 microscope (Nikon) was used to take photographs.

### RNA sequencing and analysis

To comprehensively characterize the transcriptional profile of G-MDSCs at various time points and explore whether the functional transition of G-MDSCs is linked to transcriptional modifications, G-MDSCs were sorted and sequenced. The details are described in the **Supplementary Information**. The raw data are available at GEO under accession number GSE260477 with the secure token orobwcwmbvafrcp.

### Statistical analysis

SPSS version 24 (IBM) software and GraphPad Prism software version 9 (Graphpad) were used for all analyses. Mann-Whitney U test was used to determine the difference in G-MDSCs, M-MDSCs as well as the intracellular HO-1 and IL-10 between patients with grades 0-I and grades II-IV aGvHD. The comparison of IFN-γ secretion, proliferation as well as the differentiation of Th subsets were performed using Wilcoxon signed-rank test between the control and experimental group. A *p*-value < 0.05 was considered to be statistically significant. Numerical values of *p*-values have been marked in the figures.

## Results

### G-MDSCs and intracellular HO-1 increased at day 28 in patients with aGvHD

Among the 29 patients subjected to sequential specimen collection, 11 patients developed higher grade II-IV aGvHD and 18 had no or lower grade aGvHD (grade 0 to I). 2 patients died within 100 days due to severe pneumonia and severe aGvHD, respectively. From day 28 to day 90, all patients offered specimens. However, only 7 patients consistently provided specimens on day 42,60 and 90.

**Figure 1:**
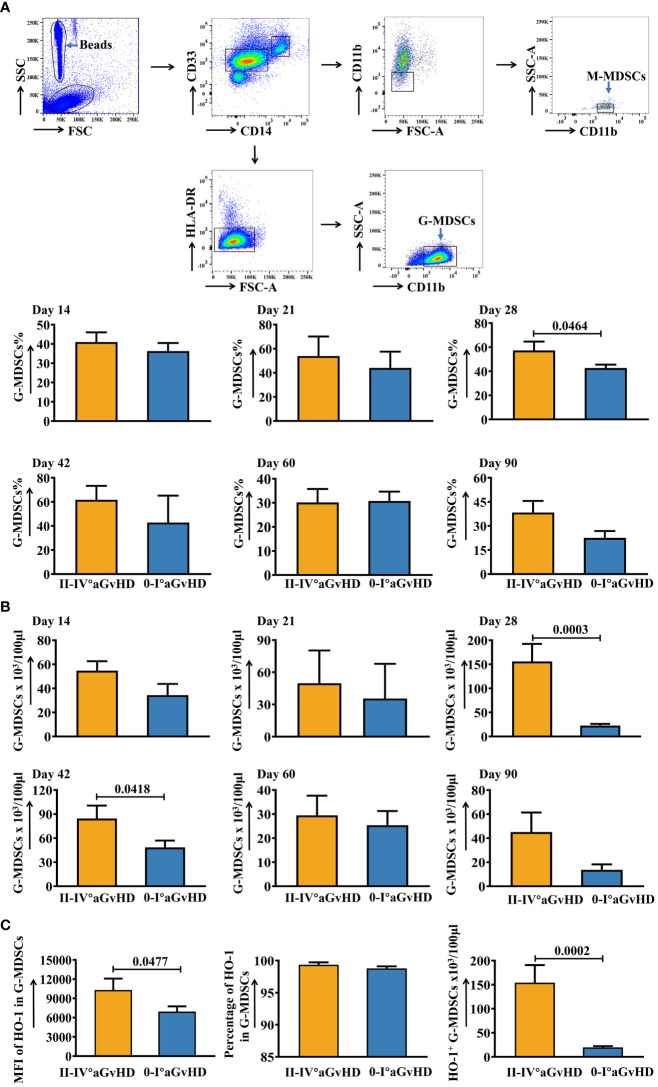
Dynamic changes of G-MDSCs in patients after allo-HSCT within 90 days. **(A)** Gating strategy of granulocytic myeloid-derived suppressor cells (GMDSCs) and monocytic myeloid-derived suppressor cells (M-MDSCs). The frequencies of G-MDSCs in patients at day 14 (n=25), day 21 (n=20), day 28 (n=17), day 42 (n=16), day 60 (n=23), and day 90 (n=21) were analyzed using flow cytometry. **(B)** The absolute numbers of G-MDSCs were calculated as well. **(C)** The mean fluorescence intensity (MFI) and percentage of intracellular heme oxygenase-1 (HO-1) in G-MDSCs at day 28 were determined using intracellular staining. The absolute number of HO-1^+^ G-MDSCs at day 28 was calculated (n=17). Bars indicate the mean value of replicates, with error bars indicating the standard error of the mean.

**Figure 2:**
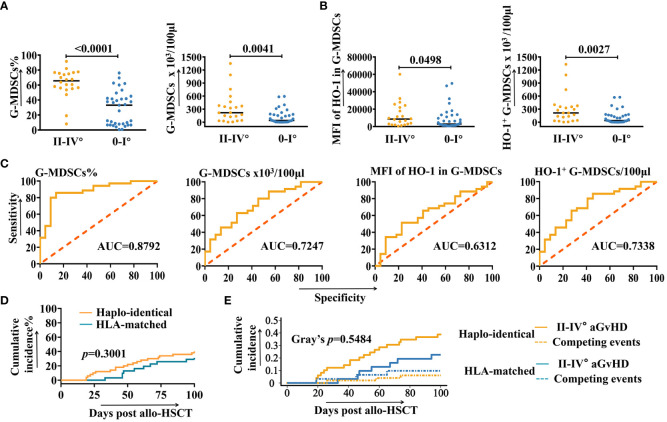
ROC and correlation analysis of G-MDSCs and intracellular HO-1. **(A, B)** The number of samples of granulocytic myeloid-derived suppressor cells (G-MDSCs) at day 28 increased to 57 and was analyzed to confirm the conclusion (n=57). **(C)** Receiver operating characteristic curves (ROC) analysis to predict the morbidity of grades II-IV aGvHD in patients after allo-HSCT at day 28, based on the number of G-MDSCs and intracellular heme oxygenase-1 (HO-1) (n=57). **(D)** Cumulative incidence of grades II-IV aGvHD in haplo-identical and HLA-matched patients. **(E)** Cumulative incidence of grades II-IV aGvHD in haplo-identical and HLA-matched patients are analyzed using a competing risks model. Competing events include deaths due to non-aGvHD causes, severe infections, and organ failures.

The gating strategy of G-MDSCs and M-MDSCs is shown in **Figure 1A**. An elevation of G-MDSCs at day 28 has been observed in patients who suffered from present/later grades II-IV aGvHD (**Figures 1A, B**). On the other hand, the percentage of M-MDSCs was higher in patients with present/later grades 0-I aGvHD (**Supplementary Figure 1**). To further study the expression of intracellular proteins due to their potential roles in immune regulation, the staining for HO-1 and IL-10 in MDSCs has been performed (**Figure 1C**; **Supplementary Figures 2**-**5**). The results showed that both the MFI of HO-1 and absolute number of HO-1^+^ cells were higher in G-MDSCs at day 28 in grades II-IV aGvHD group (**Figure 1C**). Afterwards, the number of samples has been extended to further confirm the conclusion. Similar results are shown in **Figures 2A, B**.

To assess whether the proportion of G-MDSCs and intracellular HO-1 of patients at day 28 are correlated with the higher risk of developing aGvHD, a further analysis was undertaken using receiver operating characteristic curves (ROC). As shown in **Figure 2C**, the percentage of G-MDSCs (AUC=0.8792), absolute number of G-MDSCs (AUC=0.7247), MFI of HO-1 in G-MDSCs (AUC=0.6312), and the absolute number of HO-1^+^ G-MDSCs (AUC=0.7338) were potential predictors for grades II-IV aGvHD.

**Figure 2D** displays the cumulative incidence rates of grades II-IV aGVHD for both haploidentical and HLA-matched recipients (p=0.3001). The competing risk analysis is shown in **Figure 2E**. Competing events include patient deaths due to non-aGvHD causes, as well as the severe infections and organ failures that could influence immune status and potentially trigger aGvHD. No statistically significant difference has been observed between the development of grades II-IV aGvHD and competing events (Gray’s *p*=0.5484).

### General characterization of G-MDSCs after allo-HSCT within 90 days

**Figure 3:**
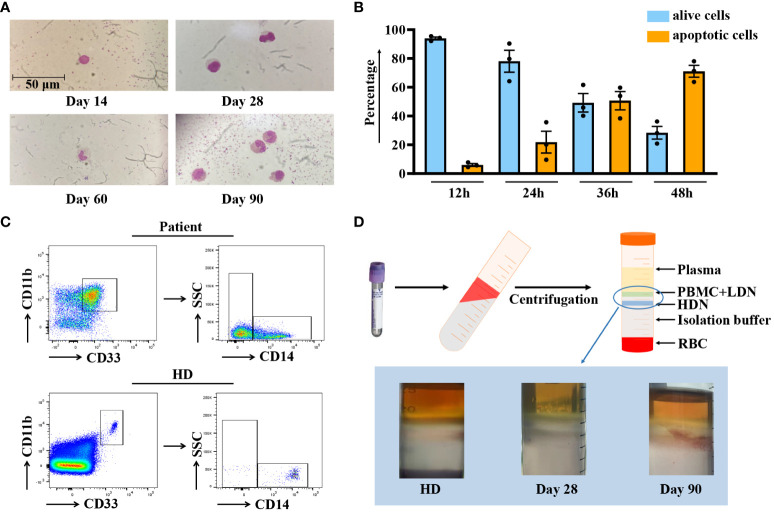
Characterization of G-MDSCs in patients within 90 days. **(A)** Wright-Giemsa stain cytospin preparations reveal the morphological characteristics of granulocytic myeloid-derived suppressor cells (G-MDSCs) across various time points. **(B)** Flow cytometry analysis was performed to determine the apoptosis ratio of G-MDSCs cultured *in vitro* at 12 h, 24 h, 36 h, and 48 h (n=3). Bars indicate the mean value of replicates with error bars indicating the standard error of the mean. **(C)** The proportion of G-MDSCs in patients was determined, after purification from fresh blood on a density gradient using Ficoll-Paque. **(D)** Leukocytes were isolated from whole blood using Polymorphprep, which separates the leukocytes into upper and lower layers based on density differences. LDN, low-density neutrophils; HDN, high-density neutrophils; RBC, red blood cells.

Before conducting functional assays, we first characterized the general features of CD11b^+^CD33^+^HLA-DR^-/low^CD14^-^ MDSCs. **Figure 3A** demonstrates that these cells exhibit a homogeneous granulocytic morphology. Therefore, we named this population CD11b^+^CD33^+^HLA-DR^-/low^CD14^-^ MDSCs with a granulocytic morphology. Subsequently, since granulocytes have a short lifespan, the G-MDSCs were stained with Annexin V/7AAD to verify the feasibility of the *in vitro* experiment. The result indicated that G-MDSCs could sustain a high survival rate for at least 24 hours (**Figure 3B**). As G-MDSCs are known as “lower density neutrophils”, the fresh blood sample from patients was isolated on a density gradient with Ficoll-paque. As expected, we noticed a significant proportion of G-MDSCs in samples from patients compared to healthy donors (**Figure 3C**). Moreover, an interesting phenomenon was observed when blood samples were isolated using Polymorphprep for all leukocytes. Compared to samples from healthy donors, those from patients exhibited a distinct distribution of cells across two layers (**Figure 3D**).

### G-MDSCs at day 28 promoted the cytokine secretion, proliferation and differentiation of autologous T cells

**Figure 4:**
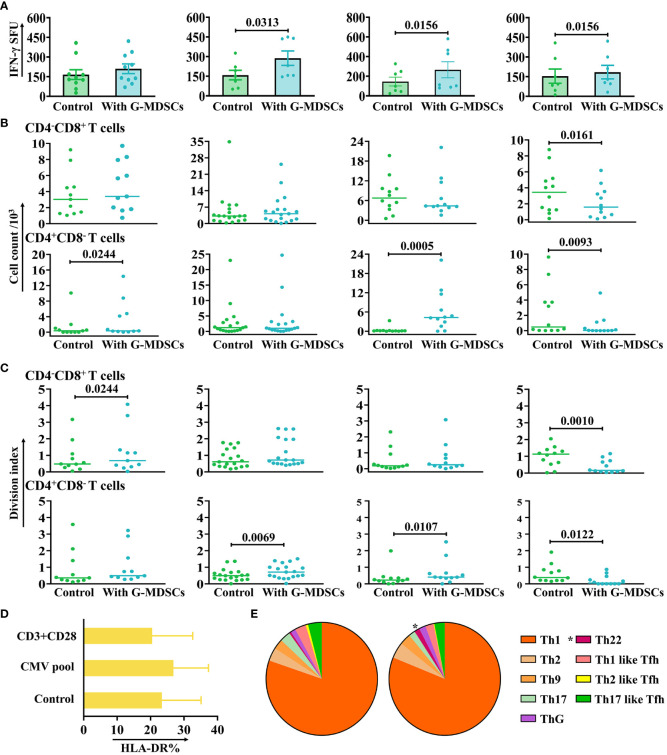
The impact of G-MDSCs on autologous T cells and NK cells. **(A)** The secretion of interferon-gamma (IFN-γ) by CMV-specific T cells in the presence of granulocytic myeloid-derived suppressor cells (G-MDSCs) at day 14 (n=10), day 28 (n=7), day 60 (n=7), and day 90 (n=7) were measured by IFN-γ ELISpot assay. Bars indicate the mean value of replicates, with error bars indicating the standard error of the mean. **(B, C)** Proliferation of CD4^-^CD8^+^ and CD4^+^CD8^-^ T cells in the presence of G-MDSCs at day 14 (n=11), day 28 (n=19), day 60 (n=12), and day 90 (n=12) were determined using flow cytometry after 4 days co-culture. Cell count and division index were determined, respectively. **(D)** G-MDSCs were co-cultured with T cells, in the presence of either the CMV pool or anti-CD3/28 for 8 hours. G-MDSCs incubated with T cells was used as control. The surface expression of HLA-DR was detected after incubation (n=11). Bars indicate mean value of replicates with error bars indicating standard error of the mean. **(E)** G-MDSCs from the patients at day 28 were co-cultured with T cells for 4 days, in the presence of anti-CD3/28. The proportion of Th subsets were evaluated using flow cytometry (n=9). *p=0.0377.

T cells have a pivotal role in the pathogenesis of aGvHD. To address the question of how G-MDSCs effect on autologous T cells, different function assays have been performed. Moreover, to minimize the variation in G-MDSCs among different patients, only samples from follow-up patients without aGvHD in the outpatient department were collected. The results showed that the IFN-γ release of CMV-specific T cells was facilitated by G-MDSCs within 90 days after allo-HSCT (**Figure 4A**). However, the proliferation of T cells in the presence of anti-CD3/28 was promoted by G-MDSCs at day 14, 28, and 60 but inhibited by G-MDSCs at day 90 (**Figures 4B, C**). Since granulocytes could acquire antigen presenting cell characteristics, we conducted the staining of HLA-DR, a pivotal MHC class II molecule responsible for antigen presentation, serves as the initial signal in immune activation. HLA-DR staining was performed following the co-culture of G-MDSCs and T cells in the presence of either CMV pool or anti-CD3/28. In comparison with the control group, the experimental group exhibited no significant changes in HLA-DR expression (**Figure 4D**). Furthermore, as G-MDSCs at day 28 promoted the proliferation of T cells, the differentiation of CD4^+^ T cells has also been determined. Data indicated that the percentage of Th22 cells was increased in the presence of G-MDSCs (*p*=0.0377) (**Figure 4E**). The gating strategy of Th cells is shown in **Supplementary Figure 6**.

### Quantity of G-MDSCs and intracellular HO-1 correlated with the function of NK cells

Concurrent with G-MDSCs immunomonitoring, NK cells were isolated from the same patients to assess their cytotoxicity. Among patients with grades II-IV aGvHD, CD107a expression on NK cells exhibited a negative correlation with the MFI of HO-1 in G-MDSCs (*p*=0.0246, r=0.4028) (**Supplementary Figure 7**), suggesting that the inflammatory state might modulate NK cell functionality.

### At day 28, G-MDSCs aggravated aGvHD and facilitated the differentiation of Th17 and Th22 cells in a xenogeneic model

**Figure 5:**
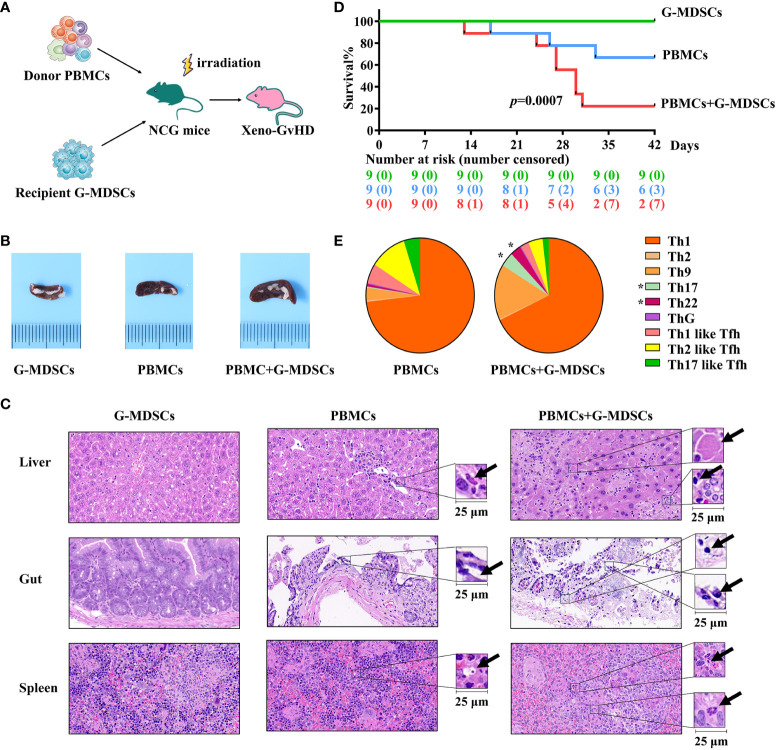
Injection of G-MDSCs from patients at day 28 into a humanized mouse model. **(A)** Protocol for establishing aGvHD model. Granulocytic myeloid-derived suppressor cells (G-MDSCs) collected from the patients at day 28 were injected into the mice with PBMCs from the related donors. **(B)** An enlarged spleen has been observed in the co-administration group. **(C)** Representative histology of the target organs harvested from the G-MDSCs alone control group, PBMC alone control group, and co-administration group. **(D)** Co-administration of G-MDSCs and PBMCs resulted in a notable decrease in the survival rate of mice (n=27). **(E)** T cell subsets were detected in the peripheral blood of mice once the proportion of human cells exceeded 20% (n=14). *p Th22 cells= 0.0313, Th17 cells=0.0469.

The protocol for the establishment of aGvHD model is shown in **Figure 5A**. As the function of T cells from patients after allo-HSCT is often inhibited by immunosuppressive medications, which may potentially lead to engraftment failure, PBMCs from patient-matched donors have been used as an alternative and avoid allogeneic reactions. Mice co-administrated with G-MDSCs and PBMCs exhibited markedly enlarged spleens, compared to those receiving G-MDSCs or PBMCs alone (**Figure 5B**). More severe tissue damage, inflammation, necrosis, and leukocyte infiltration has also been observed in the co-administered group (**Figure 5C**). Consistent with pathological findings, G-MDSCs co-administrated with PBMCs reduced the overall survival rate of mice. In parallel, administration of G-MDSCs alone did not lead to aGvHD (**Figure 5D**). Furthermore, a higher proportion of Th22 and Th17 cells in co-administrated group has been observed (*p*=0.0313 and 0.0469, respectively), suggesting G-MDSCs may facilitate the differentiation of Th22 and Th17 cells *in vivo* (**Figure 5E**).

### Differences in molecular profiling between G-MDSCs at day 28 and day 90

**Figure 6:**
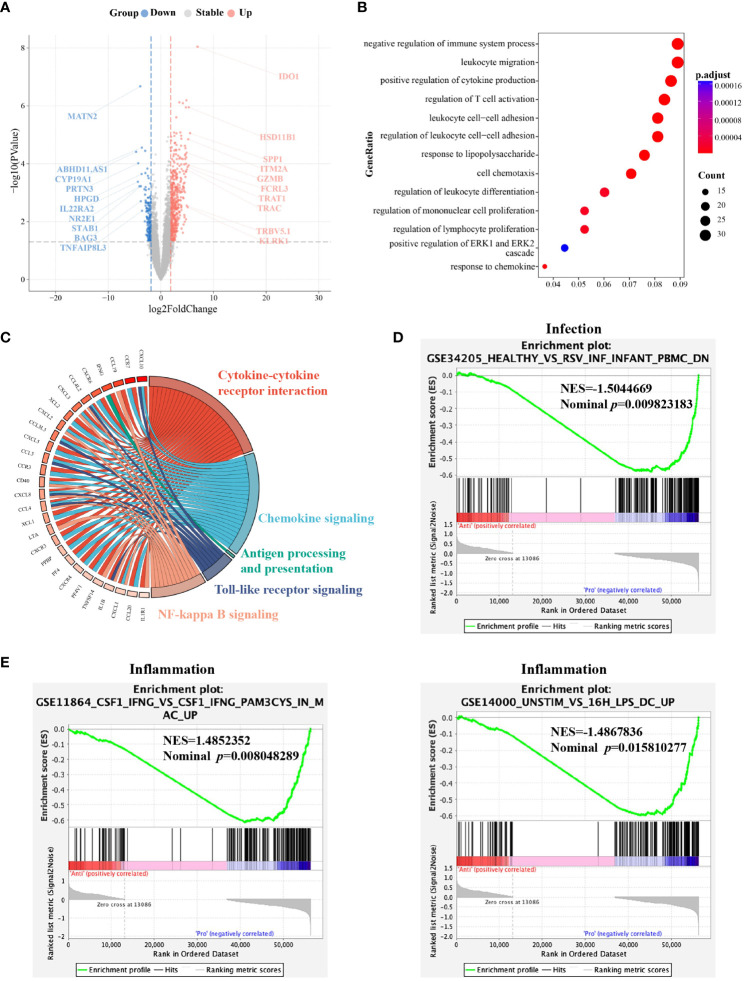
Molecular characterization of G-MDSCs differentiation as immune reconstitution progresses. **(A)** Volcano plot shows the differently expressed genes between the granulocytic myeloid-derived suppressor cells (G-MDSCs) in patients (n=7 each) at day 28 and day 90, respectively (|log2FC| >1, adjusted *p*<0.05). Red dots reveal genes expressed at a higher level at day 90. Blue dots represent the genes downregulated at day 90 compared to day 28. **(B)** Enriched GO terms were identified for elevated genes in G-MDSCs from patients at day 90 (adjusted *p*<0.05). **(C)** The GESA KEGG chord plot illustrates the connections among highly expressed genes in G-MDSCs at day 90 and their associated pathways, visually represented through connecting ribbons (NES>1, adjusted *p*<0.05). **(D, E)** GESA of genes expressed in G-MDSCs has been performed. Infection and inflammatory-associated pathways were enriched in G-MDSCs from patients at day 28.

To investigate whether the differences in the functional behavior of G-MDSCs at different time points are related to their transcriptional modulation, we sorted G-MDSCs from follow-up patients in the outpatient department at day 28 and 90 for RNA sequencing. A total of 1445 genes were differentially expressed between two-time points. The top 20 genes with the most divergent expression are shown in **Figure 6A**. Furthermore, both gene ontology (GO) enrichment analysis and gene set enrichment analysis for the Kyoto Encyclopedia of genes and genomes (GESA KEGG) revealed that genes significantly expressed in G-MDSCs at day 90 are linked to immune functional items, particularly the negative regulation of immune system process (adjusted *p*=1.16x10^-8^, q=9.98x10^-9^) and cytokine-cytokine receptor interaction pathway (NES=2.34, adjusted *p*=1.63x10^-8^, q=1.39x10^-8^) (**Figures 6B, C**). On the other hand, genes highly expressed in G-MDSCs at day 28 were enriched in the cell cycle pathway (adjusted *p*=5.81x10^-8^, q=4.97x10^-8^) (**Supplementary Table 3**). Interestingly, after we carried out GESA with all divergently expressed genes (DEG), the results showed that pathways associated with infection and inflammation were enriched in G-MDSCs at day 28 (**Figures 6D, E**).

**Figure 7:**
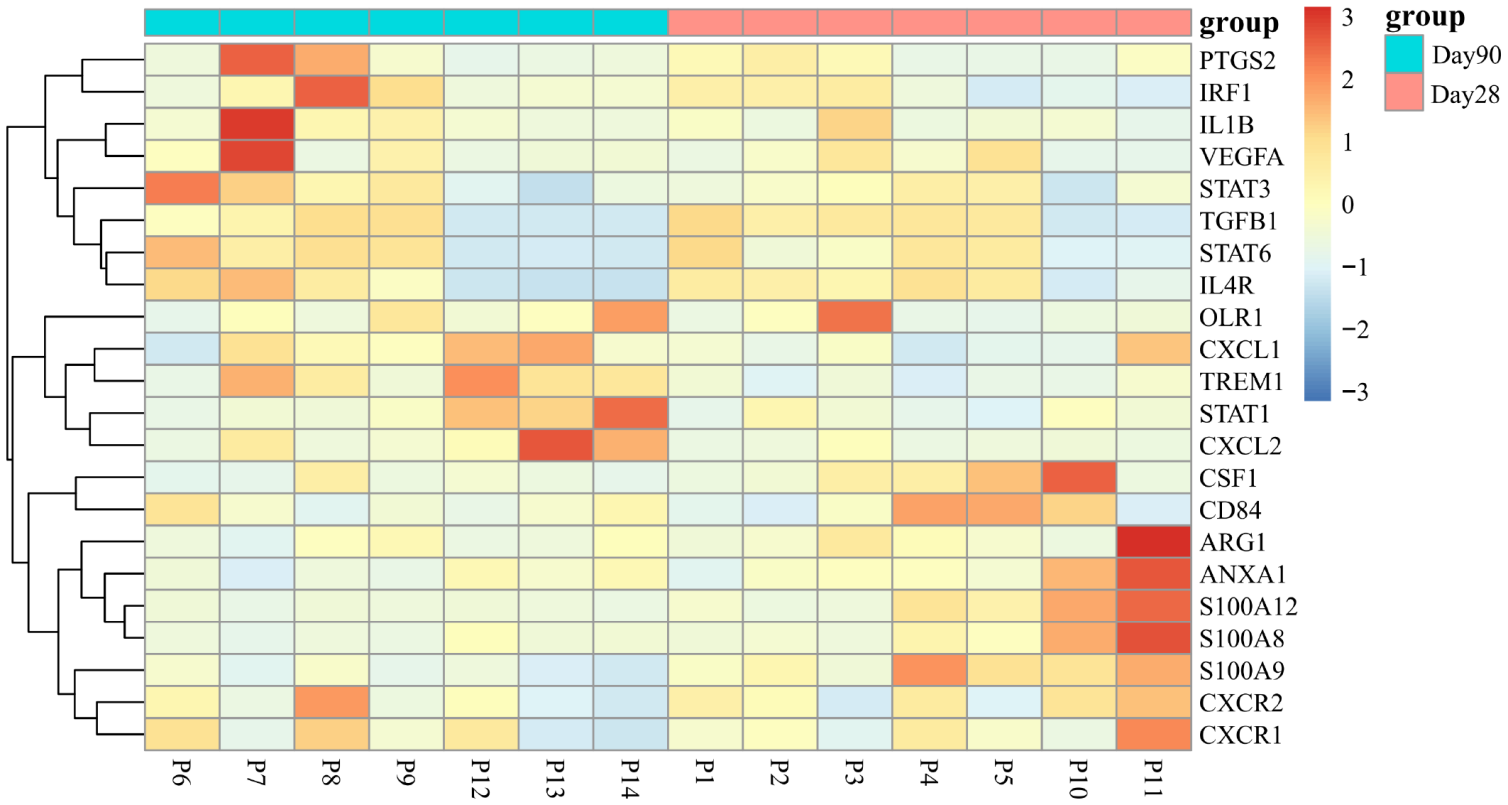
Differential expression of signature genes in G-MDSCs at day 28 and day 90. The examination assessed the expression of 22 genes, differentiating G-MDSCs from classical neutrophils, within G-MDSCs at day 28 and day 90 (|log2FC| >1, adjusted *p*<0.05).

Next, we selected 22 genes distinguishing G-MDSCs from classical neutrophils (25) to further characterize the variations between two time points. No significant difference has been found except the *CXCL2* associated with chemotaxis, was highly expressed in G-MDSCs at day 90 (log2 fold change=2.97, q=0.03) (**Figure 7**).

Recently, Paiva and colleagues reported a significant activation of the cytokine-cytokine receptor interaction pathway, during the differentiation of immature neutrophils into G-MDSCs in bone marrow (3). In line with this, our data also revealed the activation of the same pathway in G-MDSCs at day 90. Subsequently, 20 representative genes, identified as upregulated during the neutrophils to G-MDSCs transformation, were chosen for comprehensive analysis (**Supplementary Figure 8A**). Remarkably, only *CXCL2* conformed to the differential expression pattern observed in patients with multiple myeloma (**Supplementary Figure 8B**).

### ER stress may contribute to the immunosuppressive function development in G-MDSCs

**Figure 8:**
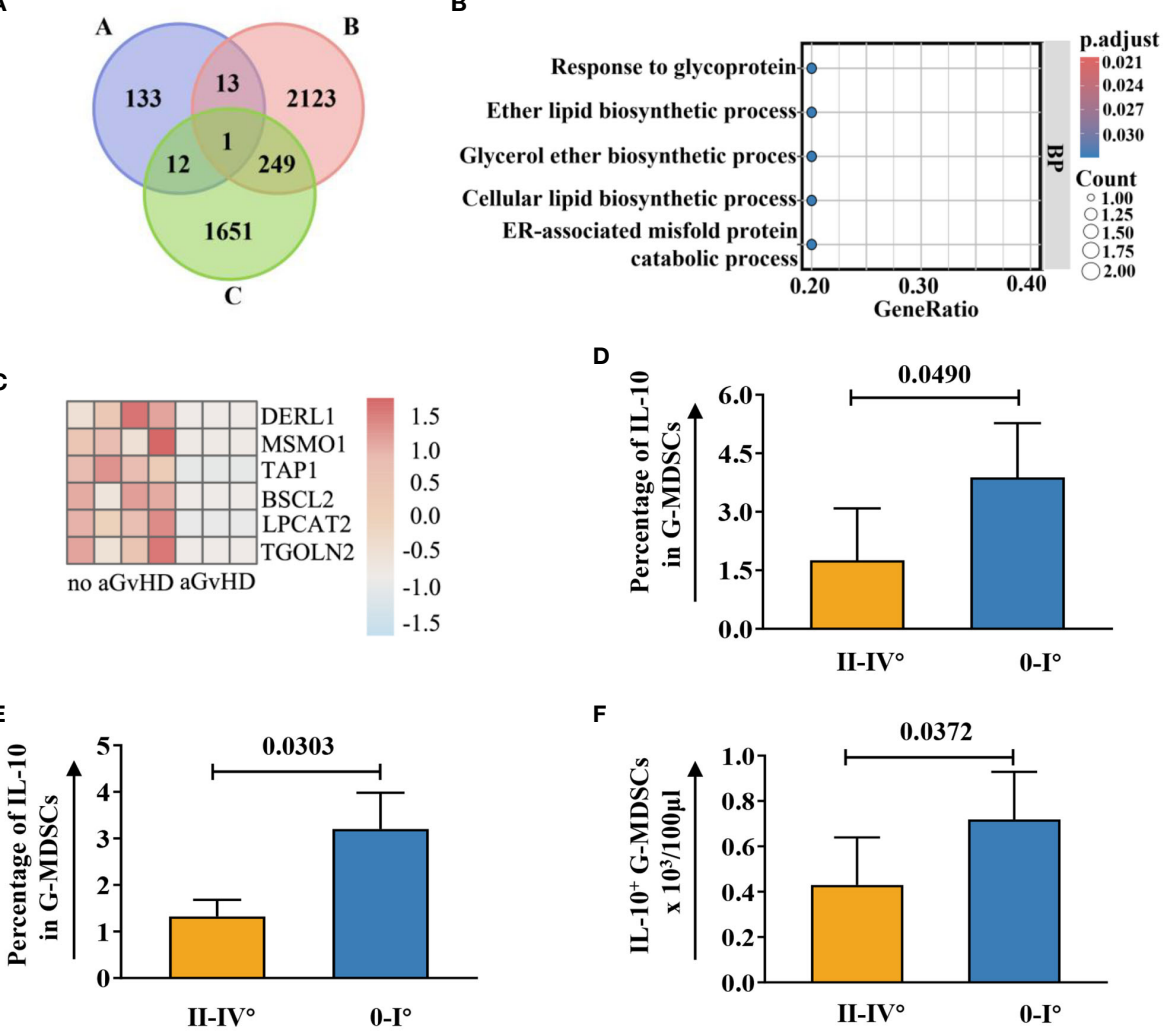
Upregulated genes associated with ER in G-MDSCs may protect patients from aGvHD. **(A)** Genetic overlap was analyzed across three cohorts: Cohort A examined genes related to heme oxygenase-1, Cohort B focused on differentially expressed genes (DEGs) in G-MDSCs comparing day 90 (n=7) to day 28 (n=4) in patients without subsequent aGvHD, and Cohort C analyzed DEGs in G-MDSCs at day 28 in patients with (n=3) versus without (n=4) subsequent aGvHD. **(B)** GO terms for intersecting genes across three G-MDSC cohorts (A-B-C, A-C, A-B intersections) were identified. **(C)** Heatmap shows DEGs associated with the endoplasmic reticulum (ER) at day 28 between patients without subsequent aGvHD (n=4) and those with (n=3). **(D)** At day 42, patients with grades 0-I aGvHD showed higher percentage of interleukin-10 (IL-10) in G-MDSCs compared to those with grades II-IV aGvHD (n=16). **(E, F)** By day 60, patients with grades 0-I aGvHD had increased IL-10 percentages and more IL-10^+^ G-MDSCs than those with grades II-IV aGvHD (n=23).

To the best of our knowledge, no study has reported on the RNA sequencing data related to G-MDSCs following allo-HSCT. Hence, we investigated key genes within G-MDSCs that confer protection against aGvHD in patients. An overlap analysis was further conducted (**Figure 8A**). Cohort A comprises genes related to HO-1 associated with anti-oxidize effect. Cohort B represents the normal immune reconstitution and studies the DEGs in G-MDSCs between patients at day 90 and day 28 without subsequent aGvHD; Cohort C examined the protective genes in aGvHD including DEGs in G-MDSCs, from patients with subsequent aGvHD beyond day 28 compared to those without. Details of overlapped genes are shown in **Supplementary Table 4**. The results revealed that the intersection of the three groups consisted of a single gene, *DERL1*, associated with endoplasmic reticulum (ER)-related degradation. After GO analysis, overlapped genes were enriched within the endoplasmic reticulum and linked to the process of protein misfolding (adjusted *p*=0.0401, q=0.0102) (**Figure 8B**).

**Figure 9:**
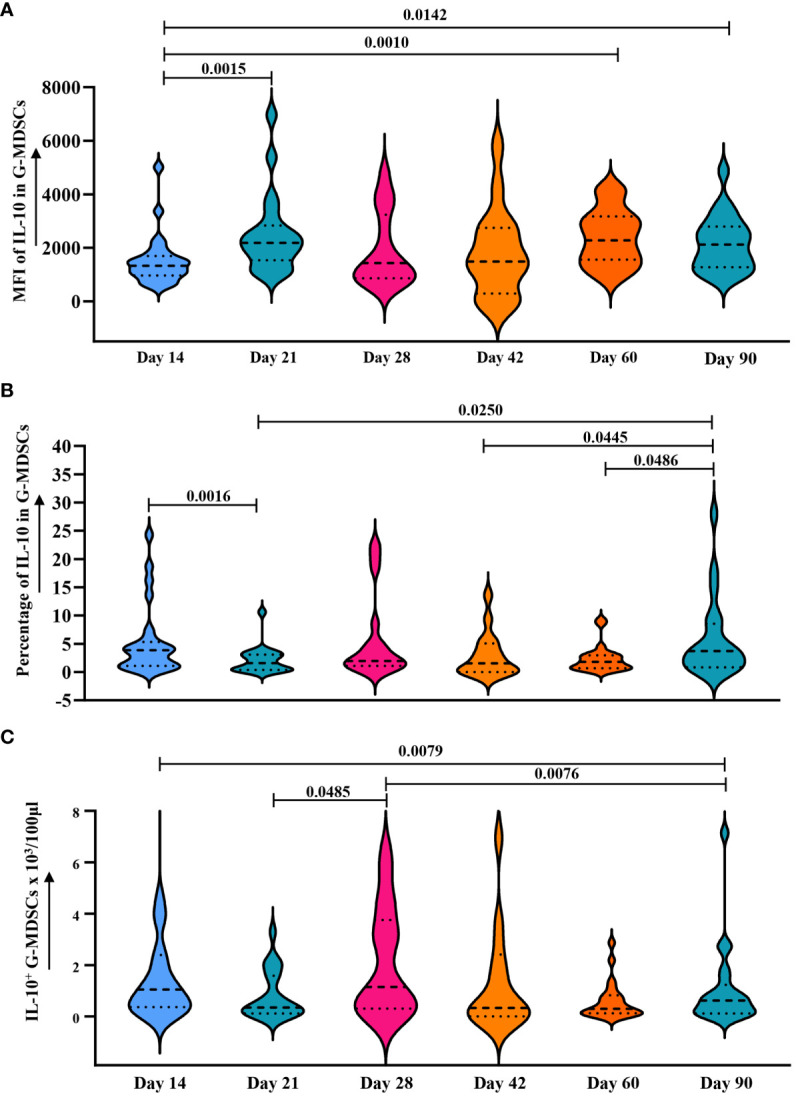
Dynamic analysis of IL-10 expression in G-MDSCs within 90 days. **(A–C)** Dynamic change of median fluorescence intensity (MFI) of IL-10 in G-MDSCs, as well as percentage of interleukin-10 (IL-10) in G-MDSCs and absolute count of IL-10^+^ G-MDSCs in patients at day 14 (n=25), day 21 (n=20), day 28 (n=17), day 42 (n=16), day 60 (n=23), and day 90 (n=21) were analyzed using flow cytometry. Dashed lines indicate the mean and quartiles of the data.

Since ER stress leads to the secretion of IL-10 in G-MDSCs (26) and genes upregulated in patients without aGvHD were correlated with endoplasmic reticulum as well (**Figure 8C**), we further analyzed the change of intracellular IL-10 in G-MDSCs within patients at different time points. No differences were detected at day 28. However, an increase in IL-10 secretion in G-MDSCs was observed at day 42 (*p*=0.0490 for percentage of IL-10 in G-MDSCs) and day 60 (*p*=0.0303 for percentage of IL-10 in G-MDSCs and *p*=0.0372 for absolute count of IL-10^+^ G-MDSCs) in patients with grades 0-I aGvHD (**Figures 8D–F**). Next, since G-MDSCs gradually acquire immunosuppressive capabilities after allo-HSCT, which is coupled with sustained stimulation from recipient antigens, potentially leads to chronic inflammation and further increased ER stress, the dynamic change of IL-10 within G-MDSCs across all time points was studied. The result shows that the secretion of IL-10 was increased in G-MDSCs at day 90 (**Figures 9A–C**).

## Discussion

Allo-HSCT is thought to be the “ultimate curative choice” for hematological malignancies in the near future (27, 28). G-MDSCs represent the major population in peripheral blood during the initial stages after transplantation. Therefore, it is essential to elucidate their properties and their association with aGvHD. It was previously assumed that G-MDSCs maintain immunosuppressive activity after allo-HSCT, similar to their function in hematologic malignancies. However, our findings suggest that increased CD11b^+^CD33^+^HLA-DR^-/low^CD14^-^ G-MDSCs at day 28 post-allo-HSCT could potentially predict the development of grades II-IV aGvHD. The present study, including *in vitro* and *in vivo* experiments, and sequencing analysis, showed that G-MDSCs promote inflammation in the early-stage post-transplantation. Subsequently, they gradually revert to their role in immunosuppression as the post-transplantation period extends.

Our findings indicate a potential correlation between aGvHD development and inflammatory status. During allo-HSCT, tissue damage caused by conditioning regimens initiates inflammation, which may increase G-MDSCs through mechanisms mediated by danger-associated molecular patterns (DAMPs) (29, 30). Hence, the excessive proliferation of G-MDSCs could be attributed to immune reconstitution and inflammatory processes. Additionally, inflammation is associated with increased oxidative stress, thereby further enhancing the upregulation of HO-1 in various cellular populations, which confers resistance to oxidative damage (31–33). Correspondingly, in patients with grades II-IV aGvHD, we observed elevated levels of HO-1, suggesting a more severe inflammatory condition.

We observed that G-MDSCs initially promote inflammation. By day 90, they developed immunosuppressive capabilities. This phenomenon might be explained by short-term inflammatory stimuli leading G-MDSCs to enhance the inflammation. However, with extended antigen exposure or persistent inflammation, G-MDSCs promote immune tolerance (3, 25). Notably, at day 90, G-MDSCs inhibited T cell proliferation without affecting cytokine secretion, a process possibly associated with indoleamine 2,3-dioxygenase (IDO) expression by G-MDSCs. A significant upregulation of the IDO gene in G-MDSCs at day 90 has been found in our study, ranking first among all DEGs. IDO, a key enzyme in tryptophan catabolism, is essential in T cell proliferation suppression and response inhibitions. Literature supports that MDSCs inhibit T cell function by enhancing IDO expression, which leads to T cell metabolism impairment by degrading tryptophan (34–36). Initially, activated T cells could secrete cytokines even under tryptophan deficient conditions. However, prolonged exposure to tryptophan depletion leads to the eventual cessation of T-cell proliferation (37).

Interestingly, our experimental findings suggest that G-MDSCs do not impair the anti-CMV immune response in patients. G-MDSCs have been found to potentially reduce the incidence of aGvHD while preserving the (graft versus leukemia) GvL effect (7). In these studies, the secretion of IFN-γ by different cells co-cultured with MDSCs, such as splenic cells, bone marrow cells, and T cells, was not affected, which may be one of the reasons for the preservation of the GvL effect (38–40).

Besides their effects on T cell proliferation and cytokine secretion, day 28 G-MDSCs promoted the differentiation of Th22 cells both *in vitro* and *in vivo*. This represents an additional mechanism through which G-MDSCs contribute to inflammation. Th22, a recently discovered CD4^+^ T cell subset, secretes specific cytokines including IL-22 and IL-13, and expresses chemokine receptors CCR4, CCR6, and CCR10 (41). Currently, the role of IL-22 in aGvHD has not reached a consensus, and donor-derived IL-22 appears to be associated with increased aGVHD (42–44). In parallel, the G-MDSCs at day 28 also promoted the differentiation of Th17 cells in the xenogeneic aGvHD model, which is another major subset of inflammatory T cells implicated in the pathogenesis of aGVHD (42).

*In vivo* experimental findings of the present study further provided evidence supporting the exacerbation of aGvHD by day 28 G-MDSCs, manifested by a decrease in survival rate and an increase in the severity of target organ damage. Moreover, spleen enlargement was observed in the co-administration group. As the initial development of MDSCs occurred in bone marrow and spleen (25), our data suggest that G-MDSCs may become activated upon co-administration with T cells.

At the transcriptional level, our findings demonstrate a significant concordance with preclinical studies. Genes markedly overexpressed in G-MDSCs at day 90 exhibited notable enrichment in pathways of NF-kappa B signaling, Toll-like receptor signaling, and cytokine-cytokine receptor interactions. Similarly, preclinical research reported an increased expression of genes associated with these pathways in G-MDSCs as well (45), which further substantiates that CD11b^+^CD33^+^HLA-DR^-/low^CD14^-^ G-MDSCs developed immunosuppressive capabilities at day 90. By contrast, cell cycle pathway associated genes were significantly upregulated at day 28. Activation of this pathway has been observed during the conversion of MDSCs into pro-inflammatory M1 pro-inflammatory macrophages within murine models (46).

In the current study, the ER stress-associated genes were upregulated in patients without aGvHD, indicating that ER stress within G-MDSCs may contribute to attenuating aGvHD. The ER, and its role in protein folding are pivotal in immune responses, impacting the antigen presentation function of dendritic cells (DCs). However, excessive ER stress leads to the conversion of DCs into MDSCs (47). Thus, ER stress may be a key trigger for initiating the immunosuppression function of MDSCs. Besides, ER stress is also associated with the lifespan, proliferation, and other characteristics of MDSCs (48).

No relevant publication has been reported on the expression of *DERL1*, a gene correlated with ER stress, in G-MDSCs following allo-HSCT. However, in immune-related disorders, this gene is associated with the severity of the disease. In rheumatoid arthritis patients, the elevated expression of *DERL1* correlates with a reduced response to infliximab treatment (49). Similarly, higher *DERL1* expression in lung adenocarcinoma and breast cancer patients is associated with poor prognosis as well (50, 51). Notably, chronic inflammation persists in these diseases, and a more severe disease state may exacerbate the inflammatory conditions, leading to increased ER stress. Likewise, persistent stimulation of recipient-derived antigens in patients undergoing allo-HSCT may induce chronic inflammation and further amplifies ER stress, thereby activating the immunosuppressive capabilities of G-MDSCs. Furthermore, Spaan and colleagues reported that increased ER stress resulted in the upregulation of the *CXCL2* gene (52), distinguishing G-MDSCs from neutrophils and conferring them with immunosuppressive functions (3, 25). Elevated expression of *CXCL2* gene in G-MDSCs at day 90 has also been observed in our study. Therefore, it can be speculated that ER stress is one of the factors contributing to the acquisition of immunosuppressive function by G-MDSCs. Upregulation of related genes, like *DERL1* and *CXCL2*, may initiate the protective effect of G-MDSCs against aGvHD and strengthen them through the secretion of ER stress-related cytokines, such as IL-10 in our study, to modulate the immune system.

Furthermore, it is worth mentioning that we additionally analyzed the general characteristics of this cell group. Regarding survival time *in vitro*, similarly to other granulocytes (53), CD11b^+^CD33^+^HLA-DR^-/low^CD14^-^ G-MDSCs exhibited a high mortality rate within 48 hours. With respect to density, comparable to other G-MDSCs, CD11b^+^CD33^+^HLA-DR^-/low^CD14^-^ G-MDSCs exhibited a significant increase in PBMCs of patients.

**Figure 10:**
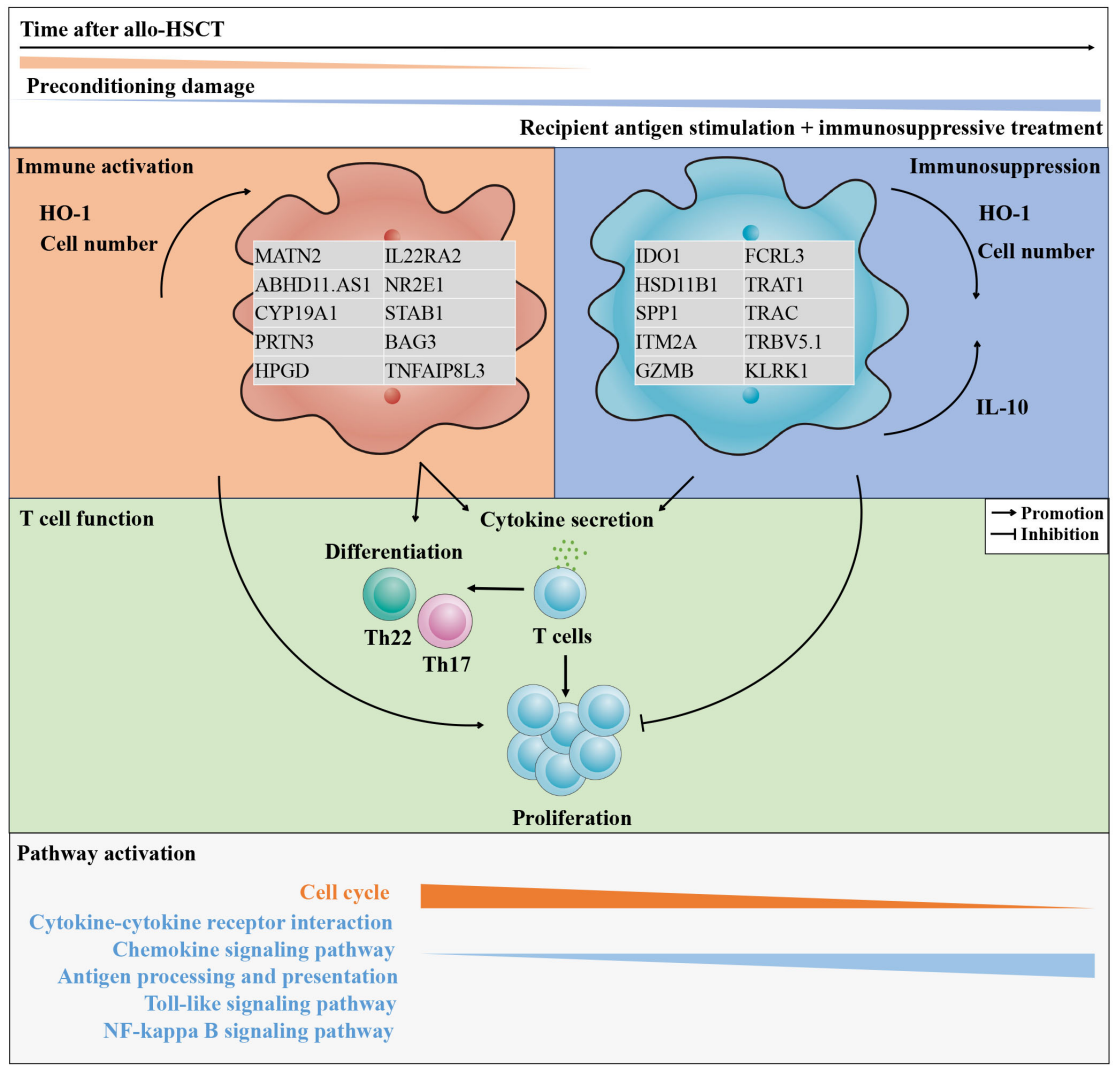
The features of CD11b^+^CD33^+^HLA-DR^-/low^CD14^-^ G-MDSCs shifted from immunostimulatory to immunosuppressive within 90 days after allo-HSCT. As the post-transplantation period lengthens, the tissue damage induced by preconditioning progressively reduces, while the impact of persistent recipient antigen stimulation and the administration of immunosuppressive medications gradually intensifies. The quantity of granulocytic myeloid-derived suppressor cells (G-MDSCs) and the intracellular oxidative stress protein, heme oxygenase-1 (HO-1), experiences a transition from an initial rise to a subsequent gradual decline. In parallel, the intracellular interleukin-10 (IL-10) increased. Throughout this process, genes with elevated expression undergo changes, accompanied by shifts in enriched pathways. Finally, by day 90, the function of G-MDSCs in promoting T cell proliferation shifts from initial promotion to an inhibitory role.

In conclusion, the present study characterized CD11b^+^CD33^+^HLA-DR^-/low^CD14^-^ MDSCs with a granulocytic morphology from patients after allo-HSCT within 90 days. The general characteristics of these cells were similar to other granulocytes. Following the preconditioning damage stimulation, CD11b^+^CD33^+^HLA-DR^-/low^CD14^-^ G-MDSCs promoted the inflammation at day 28 post-transplantation by enhancing both the cytokine secretion and proliferation of T cells, as well as their differentiation into inflammatory subsets. Data from humanized mice further confirm the hypothesis. Thus, increased G-MDSCs at day 28 may be a potential predictor for aGvHD. By contrast, persistent stimulation by recipient antigens and the sustained administration of immunosuppressive medications enables G-MDSCs to develop immunosuppressive function by day 90. Based on transcriptional data and the intracellular IL-10 level, we speculate that ER stress may contribute to the immunosuppressive function in G-MDSCs. **Figure 10** summarizes both the hypothesis and the main findings. These results may contribute to the establishment of immune tolerance after allo-HSCT and may serve as a potential predictor of aGVHD in the future. Further research is required to elucidate the cellular mechanisms that induce immune tolerance in recipients.

## Data availability statement

The datasets presented in this study can be found in online repositories. The names of the repository/repositories can be found below: GSE260477 (GEO).

## Ethics statement

The studies involving humans were approved by ethics committee of the Affiliated Hospital of Guizhou Medical University Ethics. The studies were conducted in accordance with the local legislation and institutional requirements. The participants provided their written informed consent to participate in this study. The animal study was approved by ethics committee of the Affiliated Hospital of Guizhou Medical University Ethics. The study was conducted in accordance with the local legislation and institutional requirements.

## Author contributions

MN: Conceptualization, Data curation, Formal analysis, Funding acquisition, Investigation, Methodology, Project administration, Resources, Supervision, Validation, Visualization, Writing – original draft, Writing – review & editing. JC: Data curation, Formal analysis, Funding acquisition, Investigation, Methodology, Project administration, Software, Validation, Writing – review & editing. XiY: Investigation, Methodology, Visualization, Writing – review & editing. YD: Formal analysis, Software, Visualization, Writing – review & editing. PZ: Funding acquisition, Investigation, Project administration, Writing – review & editing. TH: Investigation, Methodology, Writing – review & editing. YZ: Investigation, Methodology, Writing – review & editing. QK: Project administration, Writing – review & editing. XH: Methodology, Writing – review & editing. JZ: Methodology, Writing – review & editing. YX: Methodology, Writing – review & editing. LC: Writing – review & editing. ML: Resources, Writing – review & editing. MZ: Resources, Writing – review & editing. FZ: Resources, Writing – review & editing. SH: Resources, Writing – review & editing. YL: Resources, Writing – review & editing. XuY: Resources, Writing – review & editing. LZ: Project administration, Resources, Writing – review & editing. TZ: Resources, Validation, Writing – review & editing. BD: Resources, Writing – review & editing. BY: Resources, Writing – review & editing. DL: Investigation, Methodology, Project administration, Resources, Supervision, Validation, Writing – review & editing. JW: Supervision, Validation, Writing – review & editing.
