# Supplementary material for: Dual roles of CD11b+CD33+HLA-DR-/lowCD14- myeloid-derived suppressor cells with a granulocytic morphology following allogeneic hematopoietic stem cell transplantation: from inflammation promoters to immune suppressors within 90 days
Source: Front Immunol. 2024 Jul 8;15:1403272. doi: 10.3389/fimmu.2024.1403272 (PMC11260618; doi:10.3389/fimmu.2024.1403272)
Supplement: Supplementary file 1 [file DataSheet_1.pdf]

Supplementary Material

Patients and treatment

Patients with aplastic anemia received fludarabine, antithymocyte immunoglobulin and cyclophosphamide. Modified busulfan/cyclophosphamide were administrated into patients with hematopoietic malignances (1, 2).

The acute graft versus host disease (aGvHD) was graded according to the modified Glucksberg criteria (3). All patients received cyclosporine, mycophenolate mofetil and short-term methotrexate for GvHD prophylaxis in the first 100 days post-transplantation. The demographics are presented in Supplementary Table 2.

RNA sequencing

G-MDSCs were sorted and total RNA was extracted using Trizol reagent kit (absin) according to the protocol from manufacturer. mRNA was enriched, fragmented and reverse transcribed into cDNA using NEBNext Ultra RNA Library Prep Kit for Illumina (New England Biolabs). Afterwards, cDNA was amplified by PCR and purified. Double-stranded cDNA fragments were subjected to end-repair, and then a single 'A' nucleotide is added to the 3' ends of the blunt fragments. The product was validated on the Agilent Technologies 2100 bioanalyzer (Agilent) for quality control. Next, cDNA through quality control was sequenced using Illumina Novaseq6000 (illumina).

The raw sequencing reads were subjected to quality control using Trim-galore (Babraham Bioinformatics). The resulting high-quality reads were retained in FASTQ format for downstream analysis. The clean reads were aligned to the reference genome (GRCh38; http://genome-idx.s3.amazonaws.com/hisat/grch38_snptran.tar.gz) using HISAT2 (Johns Hopkins University). The gene expression levels were then quantified using featureCounts (Bioinformatics Research Group). Finally, a heatmap was generated using pheatmap to visualize the gene expression patterns across different samples. Differential expression analysis was performed using DESeq2 with genes exhibiting a |log2 fold change| >1 and a Q value ≤0.05 considered significantly differentially expressed. To gain insights into the biological implications of these differentially expressed genes, Gene Ontology (GO) (http://www.geneontology.org/) and Kyoto Encyclopedia of Genes and Genomes (KEGG) (http://www.kegg.jp/) enrichment analyses were performed using the hypergeometric distribution-based Phyper (http://en.wikipedia.org/wiki/Hypergeometric_distribution) tool. The significant levels of terms and pathways were corrected by Q value with a rigorous threshold (Q value ≤ 0.05) by Bonferroni.

References:

1. Yan CH, Wang Y, Wang JZ, Chen YH, Chen Y, Wang FR, et al. Minimal Residual Disease- and Graft-Vs.-Host Disease-Guided Multiple Consolidation Chemotherapy and Donor Lymphocyte

Infusion Prevent Second Acute Leukemia Relapse after Allotransplant. *J Hematol Oncol* (2016) 9(1):87. Epub 2016/09/16. doi: 10.1186/s13045-016-0319-5.

2. Ren XY, Liu X, Huang QS, Wang QM, He Y, Zhu XL, et al. Incidence, Risk Factors, and Outcome of Immune-Mediated Neuropathies (Imns) Following Haploidentical Hematopoietic Stem Cell Transplantation. *Biology of blood and marrow transplantation : journal of the American Society for Blood and Marrow Transplantation* (2019) 25(8):1629-36. Epub 2019/05/03. doi: 10.1016/j.bbmt.2019.04.021.

3. Przepiorka D, Weisdorf D, Martin P, Klingemann H, Beatty P, Hows J, et al. 1994 Consensus Conference on Acute Gvhd Grading. (1995) 15(6):825-8.

Supplementary Table 1. Patient demographics and transplantation outcomes.

Characteristic	Value
**Sex (female/male)**	41/41
**Disease**	
Acute myelogenous leukemia	40
Non-Hodgkin lymphoma	1
Myelodysplastic	6
Acute lymphoblastic leukemia	25
Severe aplastic anemia	9
Hemophagocytic syndrome	1
**Median age at transplantation, yr (range)**	33 (15-59)
**Donor-patient relationship**	
Parent-child	9
Sibling-sibling	57
Child-parent	15
unrelated donor	1
**Match status**	
HLA-matched	31
Haplo-identical	50
Others	1

**Acute GvHD**	
None or GvHD grade I	54
GvHD grades II-IV	28
**Median days to achieve ANC 500/mL (range)**	12 (8-22)
**Median $CD34^+$ stem cells transplanted, $x10^6$/kg (range)**	8.78 (4.11-18.06)

GvHD: graft versus host disease

Supplementary Table 2. Antibody list.

Name	Dye	Isotype	Clone	Company	Cat.NO.
CD33	Alexa Fluor 700	Mouse IgG1(κ)	WM53	BD	561160
CD8	PE	Mouse IgG1(κ)	4AHIT8a	4A Biotech	FHP008a-01-100
CD11b	PE	Mouse IgG1(κ)	Bear1	BECKMAN COULTER	IM2581U
CD33	FITC	Mouse IgG1(κ)	D3HL60.251	BECKMAN COULTER	IM1135U
HLA-DR	PerCP-Cy5.5	Mouse IgG2a(κ)	G46-6	BD	560652
CD14	PerCP-Cy5.5	Mouse IgG2a(κ)	M5E2	BD	550787
CD4	APC	Mouse IgG1(κ)	13B8.2	BECKMAN COULTER	IM2468U
CD14	APC	Mouse IgG1(κ)	61D3	ThermoFisher	17-0149-42
HLA-DR	APC-eFluor780	Mouse IgG2b(κ)	LN3	ThermoFisher	47-9956-42
CD11b	PerCP-Cy5.5	Mouse IgG1(κ)	LM2	Biolegend	393106
CD183	FITC	Mouse IgG1(κ)	G025H7	Biolegend	353703
CD8	APC-Cy7	Mouse IgG1(κ)	SK1	Biolegend	344713
CD45RA	Alexa Fluor 700	Mouse IgG2b(κ)	HI100	Biolegend	304119
CCR10	PE	Armenian Hamster IgG	May-88	Biolegend	341503
CD194	APC	Mouse IgG1(κ)	L291H4	Biolegend	359407

CD196	PE-Cy7	Mouse IgG2b(κ)	G034E3	Biolegend	353417
CD185	BV421	Mouse IgG1(κ)	J252D4	Biolegend	356919
CD4	BV510	Mouse IgG1(κ)	RPA-T4	Biolegend	300545
HO-1	FITC	Mouse IgG2b	HO-1-2	abcam	ab69545
IL-10	PE-Cy7	Rat IgG1(κ)	JES3-9D7	ThermoFisher	25-7108-42

Abbreviations: APC = allophycocyanin; APC-eFluor 780 = Allophycocyanin-eFluor 780; BV421 = Brilliant Violet 421; BV510 = Brilliant Violet 510; Cat. No. = catalogue number; FITC = fluorescein isothiocyanate; HO-1 = Heme oxygenase 1; PE = phycoerythrin; PE-Cy7 = phycoerythrin-Cyanin 7; PerCP = peridinin chlorophyll

Supplementary Table 3. GSEA KEGG analysis of different expressed genes in G-MDSCs between day 28 and day 90

ID	Description	Enrichment scrore	NES	Adjusted *p*	*q*
hsa04110	Cell cycle	-0.476	-2.16	$5.81x10^{-8}$	$4.97x10^{-8}$
hsa04064	NF-kappa B signaling pathway	0.457	1.62	$4.20x10^{-2}$	$3.59x10^{-2}$
hsa04620	Toll-like receptor signaling pathway	0.513	1.79	$2.83x10^{-3}$	$2.42x10^{-3}$
hsa04062	Chemokine signaling pathway	0.573	2.17	$1.00x10^{-7}$	$8.59x10^{-8}$
hsa04640	Hematopoietic cell lineage	0.591	1.95	$1.47x10^{-3}$	$1.25x10^{-3}$
hsa04612	Antigen processing and presentation	0.610	1.96	$1.47x10^{-3}$	$1.25x10^{-3}$
hsa04060	Cytokine-cytokine receptor interaction	0.613	2.34	$1.63x10^{-8}$	$1.39x10^{-8}$

Abbreviations: NES: Normalized Enrichment Score

Supplementary Table 4. Genetic overlap analysis

Gene_symbol	Description	day28 no aGvHD versus aGvHD	day28 no aGvHD versus day90	Related to endoplasmic reticulum in GEO database
DERL1	ENSG00000136986	up	up	yes
TUT1	ENSG00000149016		up	
VWCE	ENSG00000167992		up	
LRRC27	ENSG00000148814		up	
CDC20B	ENSG00000164287		up	
SLC5A5	ENSG00000105641		up	
PTGIR	ENSG00000160013		up	
SSBP2	ENSG00000145687		up	
PTGER4	ENSG00000171522		down	
RNF181	ENSG00000168894		up	
TRIM9	ENSG00000100505		up	
SLC37A4	ENSG00000137700		up	yes
CPSF1	ENSG00000071894		up	
LMX1B	ENSG00000136944		up	
CHRNB2	ENSG00000160716	down		
MSMO1	ENSG00000052802	up		yes
TNPO2	ENSG00000105576	down		

FZR1	ENSG00000105325	up	
TAP1	ENSG00000168394	up	yes
BSCL2	ENSG00000168000	up	yes
MNS1	ENSG00000138587	down	
USP7	ENSG00000187555	up	
KRAS	ENSG00000133703	up	
LPCAT2	ENSG00000087253	up	yes
TGOLN2	ENSG00000152291	up	yes
CHRNA9	ENSG00000174343	down	

Red: the overlapped genes among cohort A, B and C; Yellow: the overlapped genes between cohort B and C; Green: the overlapped genes between cohort A and C. Abbreviations: aGvHD: acute graft-versus-host disease. GEO：Gene Expression Omnibus.

Supplementary Figure 1

A

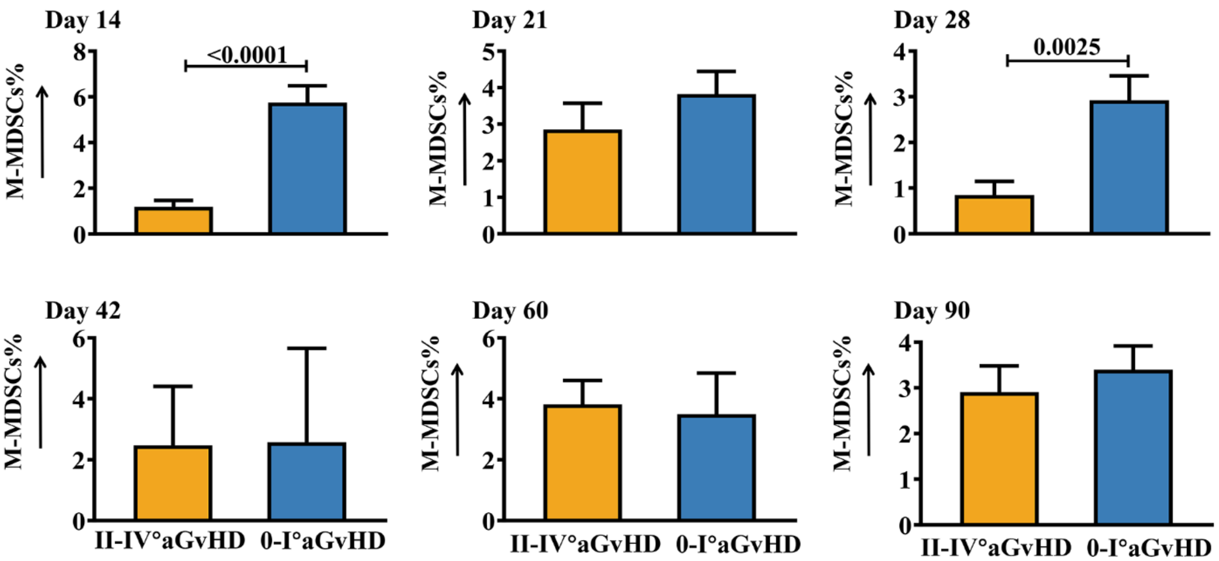

B

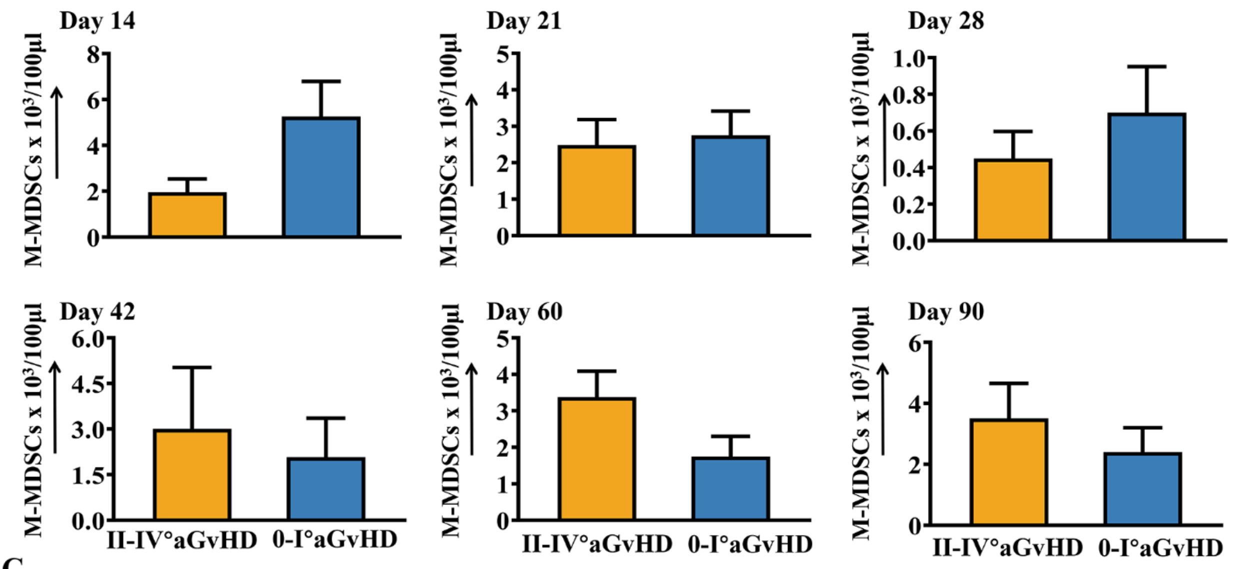

C

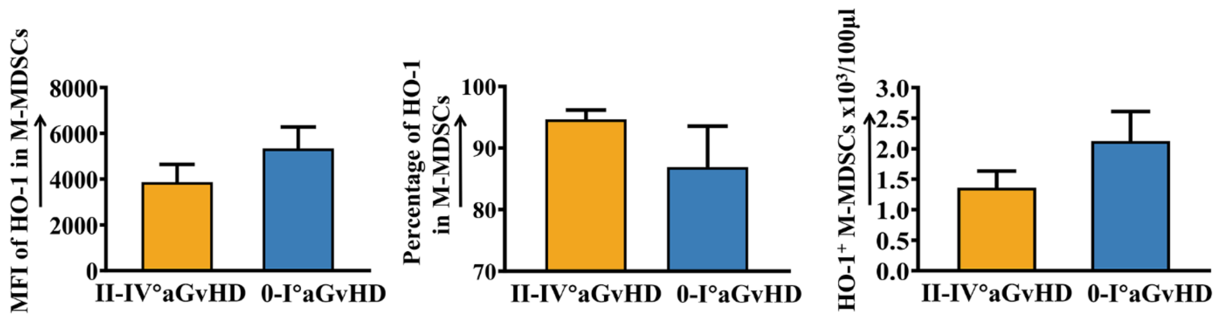

Supplementary Figure 1. Dynamic change of M-MDSCs in patients after allo-HSCT within 90 days. (A-B) The frequency and absolute number of monocytic myeloid-derived suppressor cells (M-MDSCs) in patients at day 14 (n=25), day 21 (n=20), day 28 (n=17), day 42 (n=16), day 60 (n=23), and day 90 (n=21) were analyzed using flow cytometry. (C) The mean fluorescence intensity (MFI) and percentage of intracellular heme oxygenase-1 (HO-1) in M-MDSCs at day 28 were determined using intracellular staining. The absolute number of HO-1^+ M-MDSCs at day 28 was calculated (n=17). Bars indicate the mean value of replicates with error bars indicating the standard error of the mean.

Supplementary Figure 2

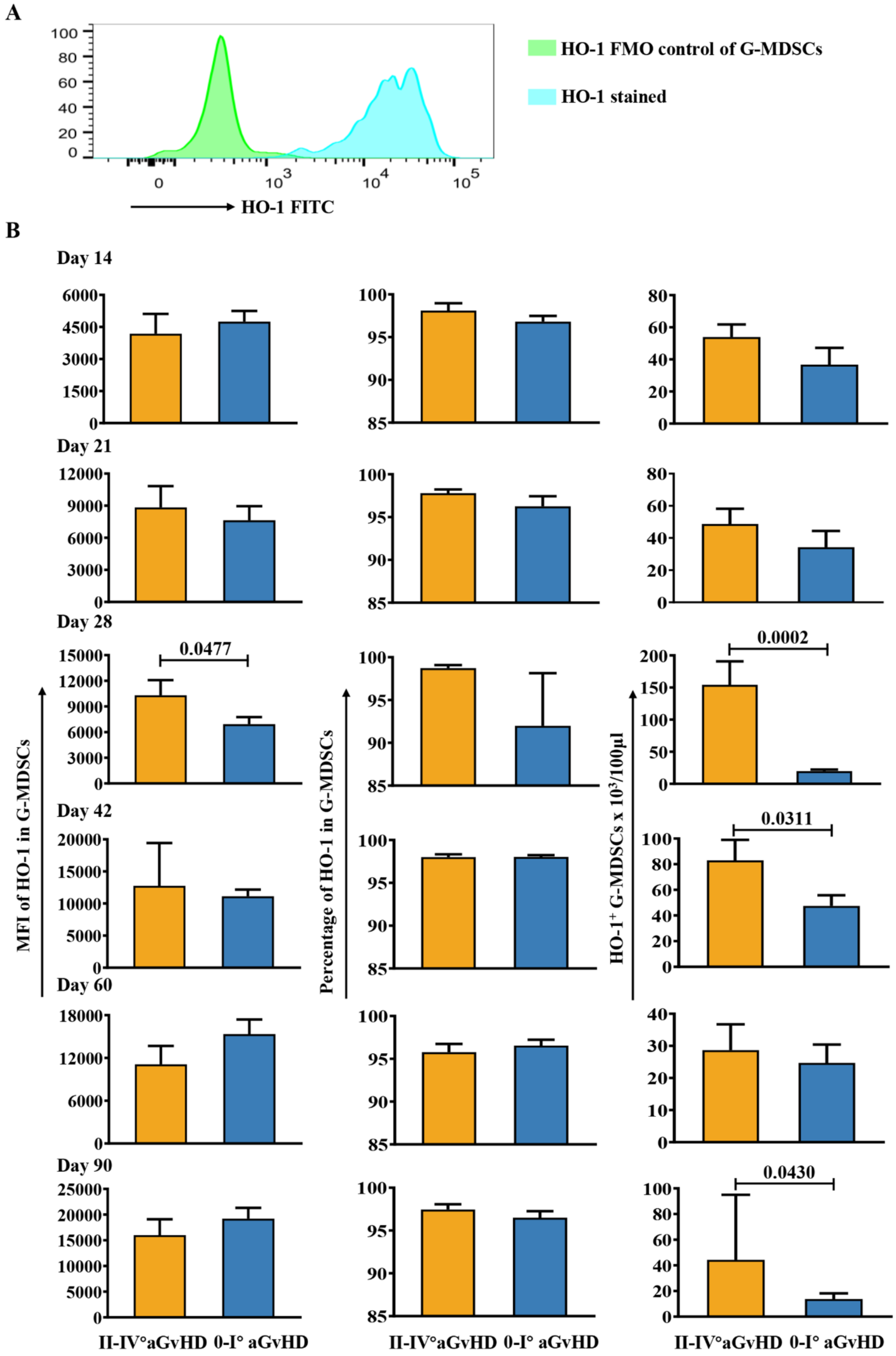

Supplementary Figure 2. Dynamic alternation of intracellular HO-1 levels within G-MDSCs were determined in patients after allo-HSCT within 90 days. (A) Gating strategy of heme oxygenase-1 (HO-1) in granulocytic myeloid-derived suppressor cells (G-MDSCs). Fluorescence minus one (FMO) staining was used as control. (B) The mean fluorescence intensity (MFI) and percentage of HO-1 in G-MDSCs, as well as the absolute number of HO-1$^+$ G-MDSCs in patients at day 14 (n=25), day 21 (n=20), day 28 (n=17), day 42 (n=16), day 60 (n=23), and day 90 (n=21) were analyzed using flow cytometry. Bars indicate the mean value of replicates with error bars indicating the standard error of the mean.

Supplementary Figure 3

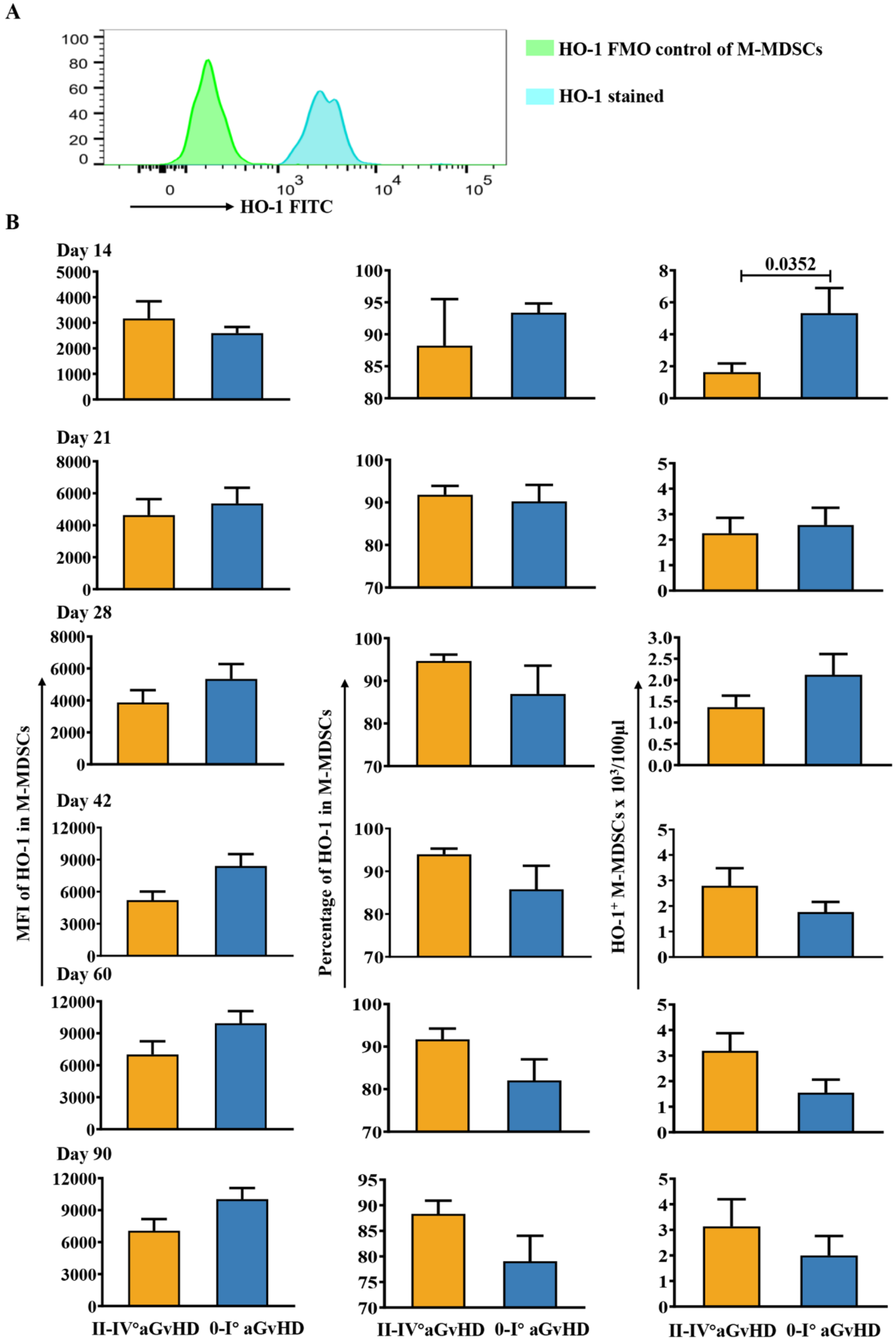

Supplementary Figure 3. Dynamic alternation of intracellular HO-1 levels within M-MDSCs were determined in patients after allo-HSCT within 90 days. (A) Gating strategy of heme oxygenase-1 (HO-1) in monocytic myeloid-derived suppressor cells (M-MDSCs). Fluorescence minus one (FMO) staining was used as control. (B) The mean fluorescence intensity (MFI) and percentage of HO-1 in M-MDSCs, as well as the absolute number of HO-1^+ M-MDSCs in patients at day 14 (n=25), day 21 (n=20), day 28 (n=17), day 42 (n=16), day 60 (n=23), and day 90 (n=21) were analyzed using flow cytometry. Bars indicate the mean value of replicates with error bars indicating the standard error of the mean.

Supplementary Figure 4

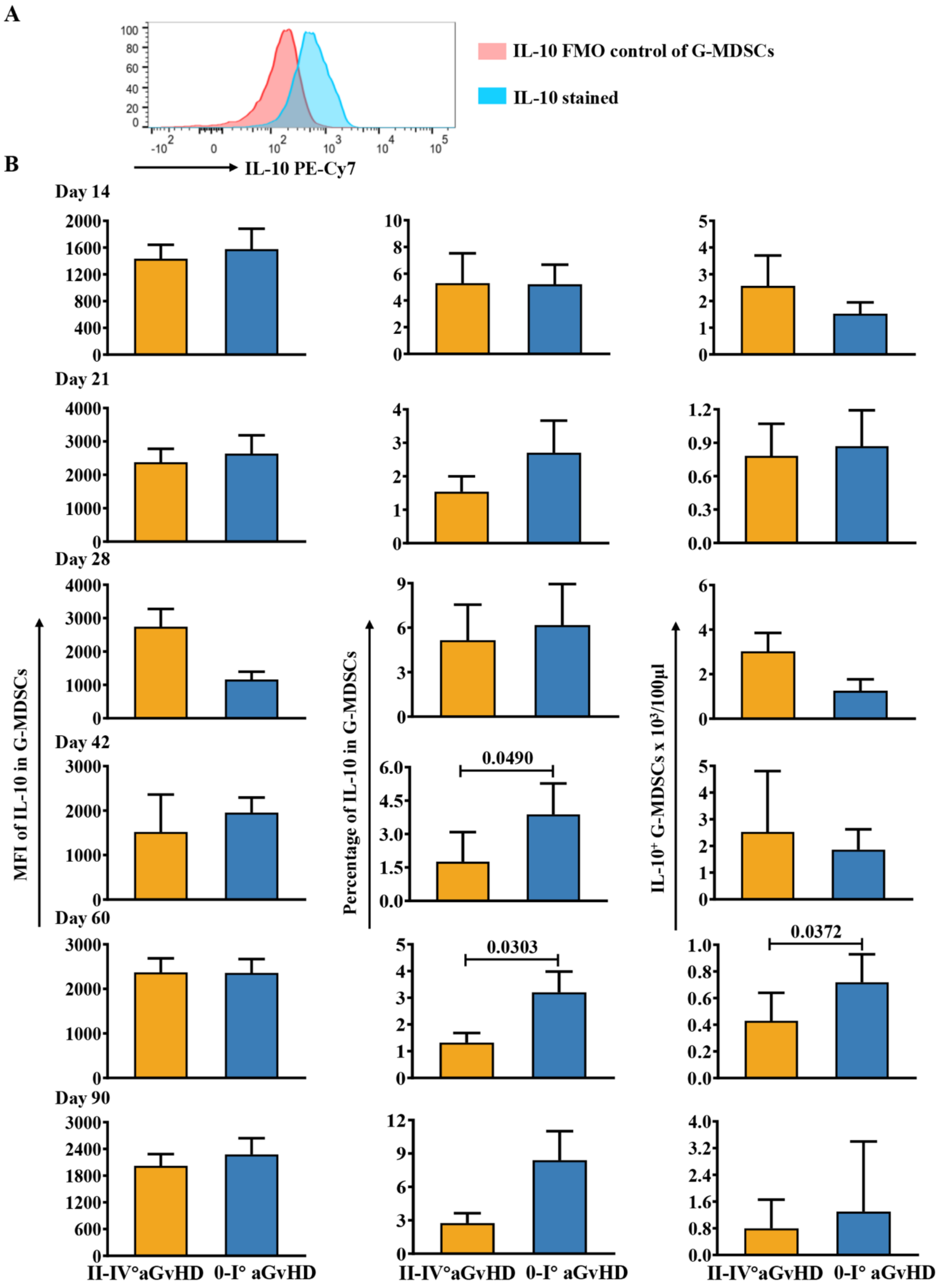

Supplementary Figure 4. Dynamic alternation of intracellular IL-10 levels within G-MDSCs were determined in patients after allo-HSCT within 90 days. (A) Gating strategy of interleukin-10 (IL-10) in granulocytic myeloid-suppressor cells (G-MDSCs). Fluorescence minus one (FMO) staining was used as control. (B) The mean fluorescence intensity (MFI) and percentages of IL-10 in G-MDSCs, as well as the absolute number of IL-10^{+} G-MDSCs in patients at day 14 (n=25), day 21 (n=20), day 28 (n=17), day 42 (n=16), day 60 (n=23), and day 90 (n=21) were analyzed using flow cytometry. Bars indicate the mean value of replicates with error bars indicating the standard error of the mean.

Supplementary Figure 5

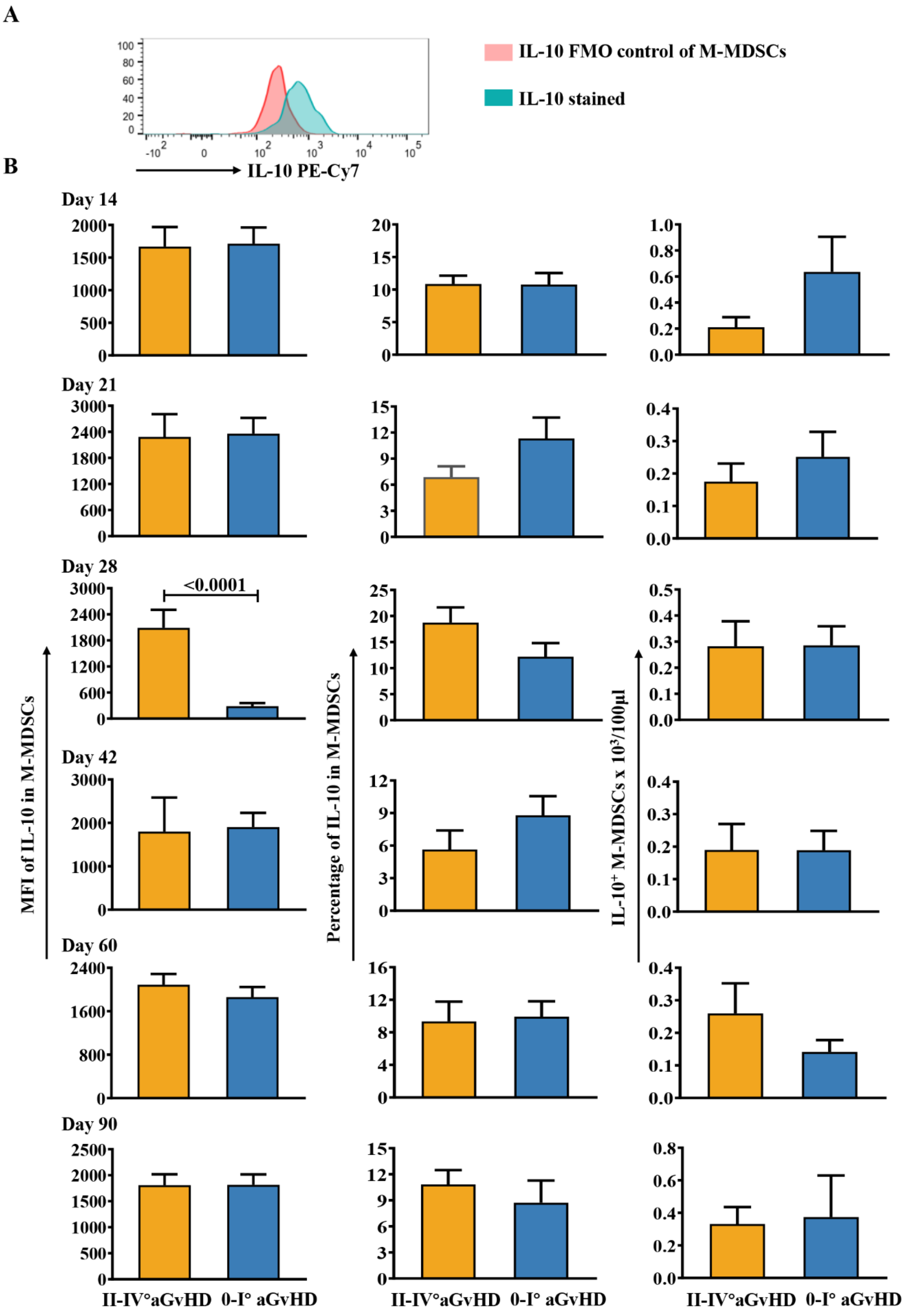

Supplementary Figure 5. Dynamic alternation of intracellular IL-10 levels within M-MDSCs were determined in patients after allo-HSCT within 90 days. (A) Gating strategy of interleukin-10 (IL-10) in monocytic myeloid-derived cells (M-MDSCs). Fluorescence minus one (FMO) staining was used as control. (B) The mean fluorescence intensity (MFI) and percentages of IL-10 in M-MDSCs, as well as the absolute number of IL-10^{+} M-MDSCs in patients at day 14 (n=25), day 21 (n=20), day 28 (n=17), day 42 (n=16), day 60 (n=23), and day 90 (n=21) were analyzed using flow cytometry. Bars indicate the mean value of replicates with error bars indicating the standard error of the mean.

Supplementary Figure 6

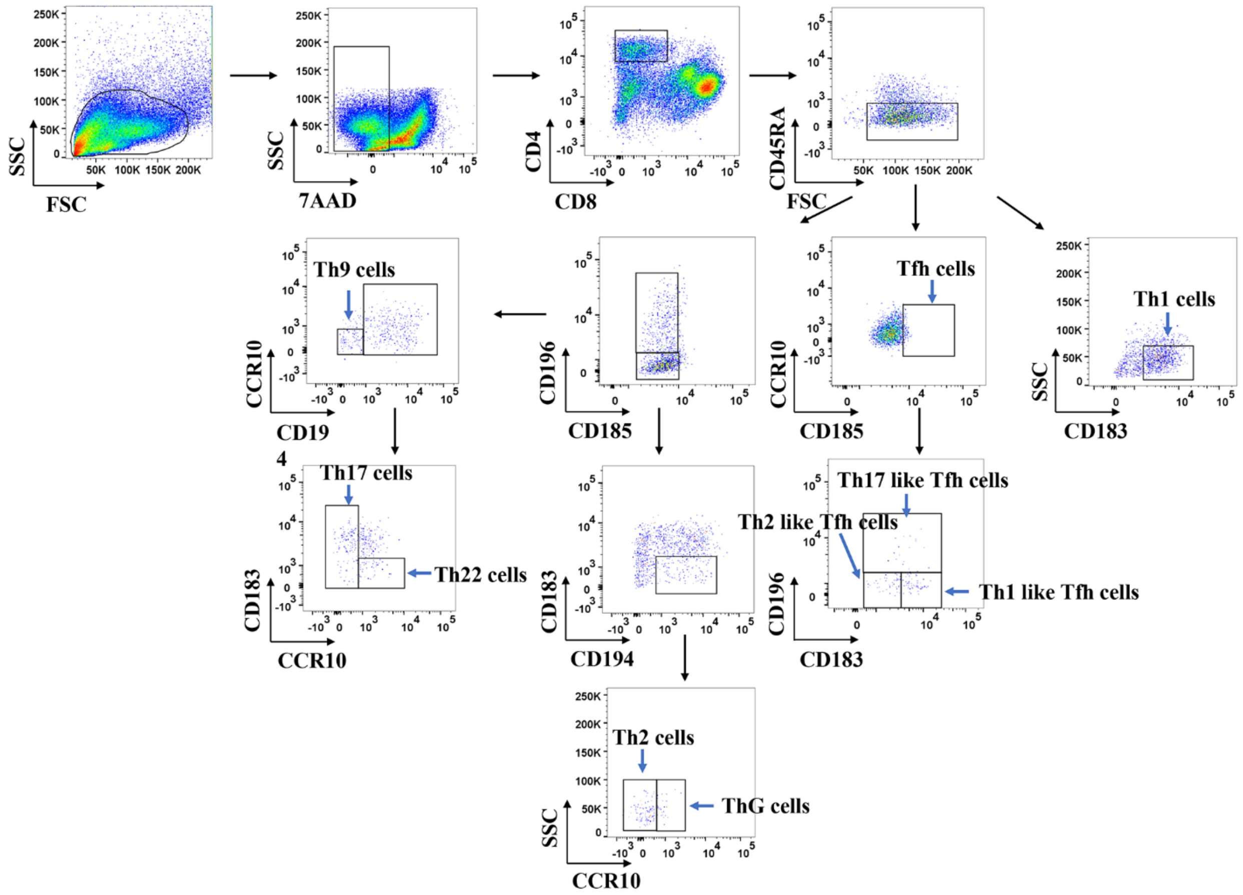

Supplementary Figure 6. Gating strategy of Th subsets. The subsets of CD4^{+} T cells were analyzed according to the expression of chemokine receptors, including Th1 (CD183^{+}), Th2 (CCR10^{-}CD194^{+} CD196^{-}), Th9 (CD194^{-}CD196^{+}), Th17 (CCR10^{-}CD194^{+}CD196^{+}), Th22 (CCR10^{+}CD194^{+}CD196^{+}), Tfh (CD185^{+}) and ThG (CCR10^{+}CD194^{+}).

Supplementary Figure 7

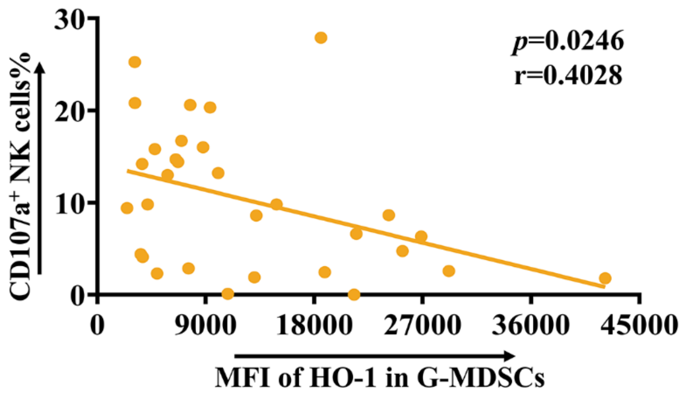

Supplementary Figure 7. Correlation analysis of NK cytotoxic function and the expression HO-1 in G-MDSCs. From day 28 to day 90, in patients with grades II-IV aGvHD, the percentage of CD107 on NK cells was negatively correlated the mean fluorescence intensity (MFI) of heme oxygenase-1 (HO-1) in autologous G-MDSC (n=31).

Supplementary Figure 8

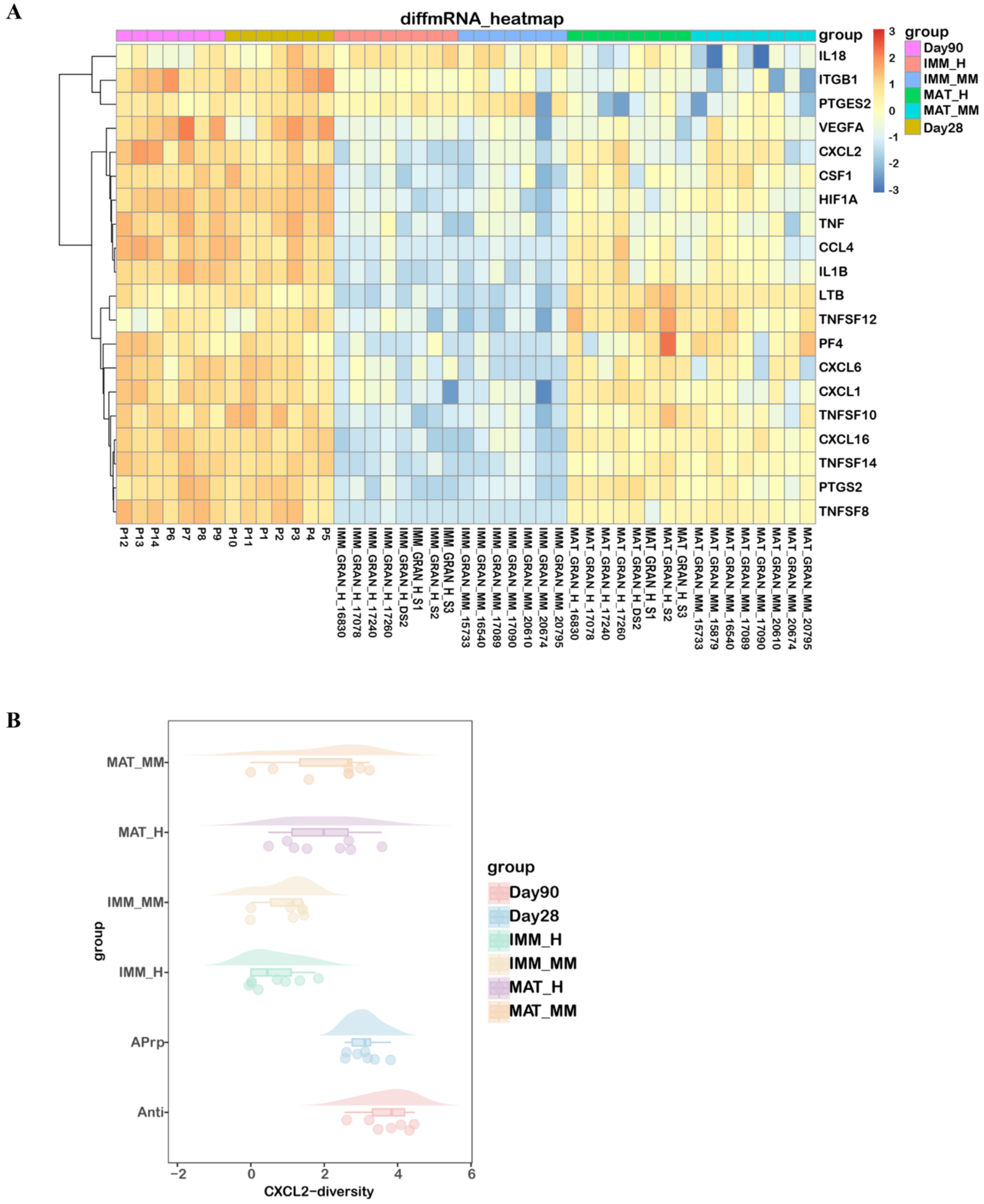

Supplementary Figure 8. Expression of genes enriched in cytokine-cytokine receptor interaction pathway in G-MDSCs (A) Based on our results and sequencing data from publication (GEO accession: GSE150021), we analyzed the expression of 20 genes that enriched in cytokine-cytokine receptor interaction pathway, which distinguish immature neutrophils from mature granulocytic

myeloid-derived suppressor cells (G-MDSC) in patients with multiple myeloma. (B) The expression of *CXCL2* was higher in G-MDSCs at day 90, which conformed to the differential expression pattern in patients with multiple myeloma. Abbreviations: IMM_H: immature neutrophils from healthy donors; IMM_MM: immature neutrophils from multiple myeloma patients; MAT_H: mature neutrophils/G-MDSCs from healthy donors; MAT_MM: mature neutrophils/G-MDSCs from multiple myeloma patients.
